# Spatiotemporal Trend of PFAS in Estuarine Sediments: Insights into Chlorinated Polyfluoroalkyl Ether Sulfonate Transformation

**DOI:** 10.1021/acs.est.5c02731

**Published:** 2025-04-02

**Authors:** Qi Wang, Yuefei Ruan, Yetong Shao, Linjie Jin, Naiyu Xie, Xiaoqiang Yang, Yuanyuan Hong, He Wang, Akira Tsujimoto, Moriaki Yasuhara, Kenneth Mei Yee Leung, Paul K. S. Lam

**Affiliations:** † State Key Laboratory of Marine Pollution (SKLMP), 25809 City University of Hong Kong, Hong Kong SAR 999077, China; ‡ Research Centre for the Oceans and Human Health, City University of Hong Kong Shenzhen Research Institute, Shenzhen 518057, China; § 26469 Southern Marine Science and Engineering Guangdong Laboratory (Zhuhai), Zhuhai 519080, China; ∥ Department of Chemistry, City University of Hong Kong, Hong Kong SAR 999077, China; ⊥ School of Biological Sciences, Area of Ecology and Biodiversity, Swire Institute of Marine Science, Institute for Climate and Carbon Neutrality, and Musketeers Foundation Institute of Data Science, The University of Hong Kong, Hong Kong SAR 999077, China; # Institute of Education, Academic Assembly, 12938 Shimane University, Matsue, Shimane 690-8504, Japan; ∇ School of Energy and Environment, City University of Hong Kong, Hong Kong SAR 999077, China; ○ Department of Applied Science, School of Science and Technology, Hong Kong Metropolitan University, Hong Kong SAR 999077, China

**Keywords:** Cl-PFESA, F-53B, H-PFESA, PFECHS, temporal trend, sediment core

## Abstract

Per- and polyfluoroalkyl substances (PFAS) are synthetic long-lasting chemicals. Marine sediment is a major repository for PFAS in the environment; accordingly, this work investigated 45 legacy and emerging PFAS in samples of surface sediments and sediment cores (1940s–2020s) collected in the Pearl River outlets, its estuary, and the adjacent northern South China Sea (NSCS), one of the global pollution hotspots. The range of total PFAS concentrations in surface sediments from the river outlets and the NSCS was 244–14400 pg/g dry weight (dw) and 31.6–363 pg/g dw, respectively. In sediment cores, perfluorooctanesulfonate (PFOS) concentrations initially increased and then declined around ten years ago. Levels of long-chain perfluorinated carboxylates have been increasing since the 1980s and experienced an accelerated rise in the 2000s. Hydrogen-substituted polyfluoroalkyl ether sulfonate (H-PFESA) was widely found in sediment samples for the first time. The ratios of 6:2 H-PFESA to 6:2 chlorinated (Cl-) PFESA in sediment cores exceeded those in surface sediment and exhibited an increasing trend with the sediment age, implying the gradual transformation of 6:2 Cl-PFESA to its hydrogen-substituted analog in sediments. A preliminary risk assessment indicated that ∑6:2 PFESAs and PFOS posed medium to high risks over recent decades.

## Graphical abstract

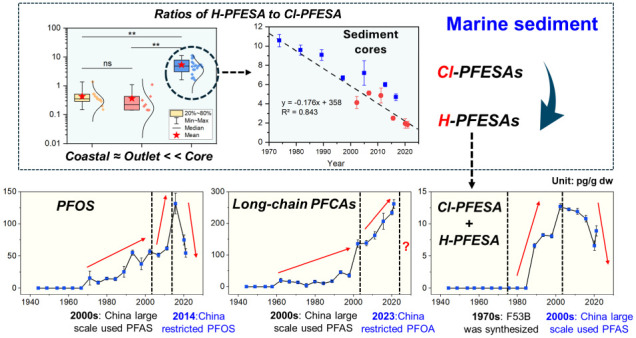

## 1 Introduction

Per- and polyfluoroalkyl substances (PFAS) are synthetic organofluorine chemicals with remarkable biochemical stability and exceptional surface activity, widely used for over 70 years.^1,2^ Among them, perfluoroalkyl acids (PFAAs) have attracted substantial attention from the scientific community, especially since the early 2000s, because of their persistent nature, bioaccumulation potential, toxic properties, and long-range transport capability.^3,4^ Certain PFAAs, including perfluorooctanesulfonate (PFOS), perfluorooctanoate (PFOA), and perfluorohexanesulfonate (PFHxS), have been recognized as persistent organic pollutants (POPs), and listed under the Stockholm Convention for global restriction or elimination.^5–7^ Besides these well-known PFAS, there is a wide array of PFAS with structures and properties similar to PFAAs, commonly referred to as emerging PFAS. These PFAS may have been used for decades, but they have not received much attention until the past decade, owing to the continuous improvement in analytical techniques and the increasing awareness of PFAS pollution.^8–10^ One representative emerging PFAS is perfluoroethylcyclohexanesulfonate (PFECHS), an analog and replacement for PFOS, which is mainly used as an erosion inhibitor in aircraft hydraulic fluids.^11^ Its presence in the environment was first reported in 2011.^9^ Another representative is 6:2 chlorinated polyfluoroalkyl ether sulfonate (Cl-PFESA), used in the metal plating industry in China since the 1970s.^8^ However, its environmental occurrence was not revealed until 2013, and currently, there is no regulatory policy for Cl-PFESA in China.^8^ In 2017, transformation products of Cl-PFESAs, named hydrogen-substituted PFESAs (H-PFESAs), were identified for the first time in the environment.^12^ Many studies have highlighted the widespread presence of emerging PFAS in various environmental and biological samples. However, compared to the vast documentation of legacy PFAS, little is known about the historical variations in the production and use of emerging PFAS.

As PFAS have relatively high water solubilities compared with other persistent contaminants, the aquatic environment plays a crucial role in PFAS transportation.^10,13^ PFAS can be readily transported via runoff from the river outlets to the adjacent seas (and ultimately oceans).^10,13,14^ PFAS in industrial waste, landfill leachate, and wastewater effluent can eventually enter coastal waters, resulting in the ubiquitous occurrence of PFAS in the marine environment.^13,15,16^ PFAS in coastal waters can partition to suspended particles, leading to their transport and deposition in marine sediments.^17^ As a result, marine sediments serve as one of the primary sinks for PFAS.^2^ Through sediment desorption and benthos bioaccumulation, PFAS deposited in sediments can be released back into the aqueous phase via resuspension, making sediments a potential source of aquatic PFAS contamination.^18–20^ PFAS have been widely found in surface sediments worldwide. However, related studies focusing on emerging PFAS are still limited, calling for further investigation to deepen our understanding of the behavior of emerging PFAS in sediment-associated environments.^20,21^ Apart from surface sediments, sediment cores, which can serve as valuable archives for studying the distribution and history of PFAS, have been mainly used to reflect the past pollution trend of PFAAs. For instance, PFAA concentrations in sediment cores collected from semienclosed bays in Korea showed an apparent increase since the 1980s.^22^ PFOS concentrations in sediment cores collected from Puget Sound, Washington, USA, exhibited an increasing trend since the 1970s.^23^ Nonetheless, historical records about the chronologically long-term occurrence of emerging PFAS have been scarcely studied.

The Guangdong–Hong Kong–Macao Greater Bay Area (GBA) is located within the Pearl River Estuary (PRE) and is considered one of the most highly developed regions in China. The northern South China Sea (NSCS), which is adjacent to the PRE, is highly influenced by PFAS discharged from the GBA via the eight major outlets of the Pearl River.^24,25^ Previous studies have reported the widespread occurrence of emerging PFAS in seawater and marine organisms in the NSCS, for example, Cl-PFESAs, H-PFESAs, PFECHS, and *p*-perfluorous nonenoxybenzenesulfonate (OBS).^25,26^ Notably, 6:2 Cl-PFESA in marine mammals from the NSCS have been reported at levels of micrograms per gram dry weight (μg/g dw), which is among the highest levels observed in biological tissue samples globally;^26^ in the same study, using high-resolution mass spectrometry, 18 Cl- and H-PFESA analogs were identified, and 15 of these emerging PFAS were seldom reported in the marine environment before.^26^ Cl- and H-PFESAs were also widespread in the seawater, fishes, crustaceans, and mollusks from the PRE where 6:2 and 8:2 Cl-PFESAs were found to exhibit comparable or even higher biomagnification potential than PFOS, indicating the noteworthy ecological risk of these emerging PFAS.^25,27^ Nevertheless, the current inventory of emerging PFAS in sediments from the PRE remains insufficiently understood. Moreover, the historical applications of emerging PFAS in China, as well as on a global scale, are not well documented.

The rapid industrialization and urbanization in the GBA have resulted in a substantial release of PFAS into the surrounding environment, making it one of the most representative regions of PFAS contamination in China and globally.^28,29^ Conducting a comprehensive investigation into PFAS in this region, with consideration of both spatial and temporal distributions, can provide critical information for marine conservation efforts. Such an investigation can also offer insights into the environmental fate and historical application of emerging PFAS in the marine environment, thereby contributing to the understanding of the current and historical ecological risks posed by PFAS to coastal ecosystems. The main objective of this study was to provide firsthand data on the horizontal and, more importantly, vertical distribution of legacy and emerging PFAS in the PRE sediments. By analyzing the sediment cores with a dated span of approximately 80 years, we aimed to characterize the historical dynamics of legacy and emerging PFAS released from the GBA, China, shedding new light on PFAS environmental issues, and setting a contemporary baseline/benchmark for PFAS regulatory considerations.

## 2 Materials and Methods

### 2.1 Chemicals and Reagents

Detailed information on the studied PFAS analytes is listed in Text S1 and Table S1. Milli-Q water was used throughout the experiment (Millipore; Bedford, USA). HPLC-grade methanol and acetonitrile were purchased from Merck (Darmstadt, Germany). The ammonium acetate (for iron analysis, purity 97%) was purchased from Wako Pure Chemical Corporation (Osaka, Japan).

### 2.2 Sediment Collection, Dating, and Total Organic Carbon (TOC) Analysis

Surface sediment samples were collected in the eight major Pearl River outlets (*n* = 8; labeled O1–O8 from west to east) and the NSCS (*n* = 22) in 2020 (Figure 1). Four sediment cores were collected within the PRE: one from Lingdingyang near western Hong Kong waters, which have relatively strong estuarine characteristics such as low salinity (core LD, approximately 61 cm in length), one from Deep Water Bay in southern Hong Kong waters, which has comparatively high clarity and salinity (core DWB, approximately 86 cm in length), one from Tolo Harbour in eastern Hong Kong waters (core TLH1C, approximately 82 cm in length), and one from Deep Bay in western Hong Kong waters (core DB2C, approximately 117 cm in length). Each sediment core was evenly sectioned into 12, 19, 11, and 12 slices, respectively, which were then dated using ^210^Pb excess and ^137^Cs techniques, and the Constant Initial ^210^Pb Concentration (CIC) model was applied to predict the deposition year of each sediment core slice, where each data point denoted a date representing the midpoint of each slice.^30,31^ Total organic carbon (TOC) was determined using the solid combustion method. Detailed information is listed in Text S2, Tables S2, S3, and Figure S1.

**Figure 1:**
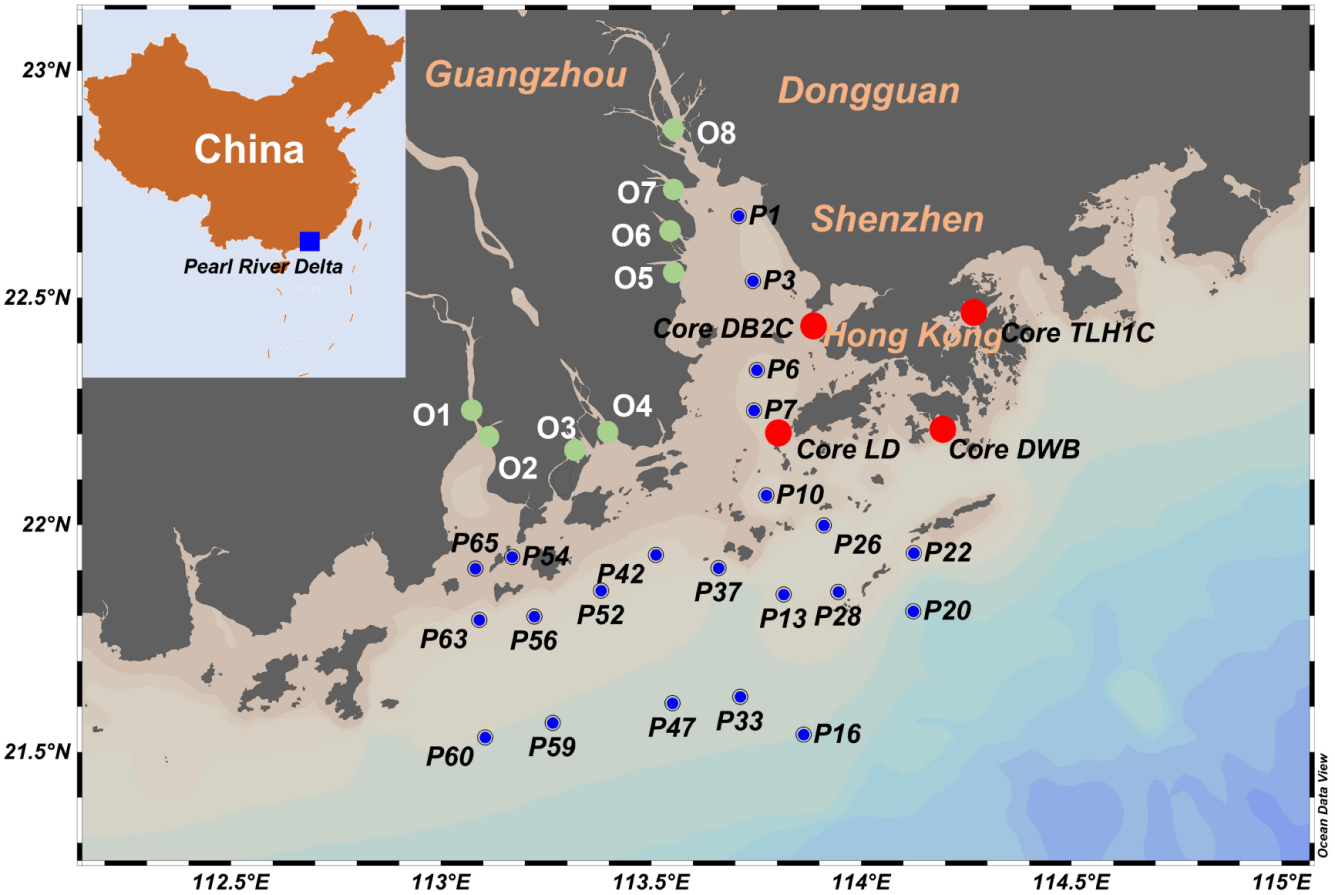
Sampling sites of surface sediments and sediment cores.

### 2.3 Sample Treatment and Instrumental Analysis

Sample extraction and cleanup methods were performed following the procedures used in our previous studies.^24,32^ Briefly, 1.0–1.5 g of homogenized freeze-dried sediment samples were extracted using sonication in methanol and purified using ENVI-Carb cartridges (250 mg, 3 cc; Supelco, Bellefonte, USA). The eluates were concentrated to 0.5 mL under a gentle stream of high-purity nitrogen prior to instrumental analysis.

Instrumental analysis of PFAS was performed following the procedures used in our previous study,^25^ using an Agilent 1290 Infinity ultraperformance liquid chromatograph (UPLC) (Palo Alto, CA, USA) interfaced with a Sciex 5500 QTRAP tandem mass spectrometer (MS/MS) with negative electrospray ionization (ESI) (Foster City, CA, USA), operating under the multiple reaction monitoring mode. Detailed information on the sample treatment and instrument analysis is provided in Text S3 and Table S4.

### 2.4 Quality Assurance and Quality Control

The matrix-spiked recoveries (*n* = 3) of individual native PFAS ranged from 58% to 96% (Text S3 and Table S5). Each batch of 10 samples contained one empty tube as a procedural blank. The instrument quantification limit (IQL) was defined as an instrumental signal-to-noise ratio of 10:1. If the analyte was not found in the procedural blank samples, the method quantification limit (MQL) was calculated using the IQL divided by the matrix concentration factor. Otherwise, MQL was defined as the average concentration of the procedural blank plus three times their standard deviation, then divided by the matrix concentration factor (Table S5). Surrogate standards were added before sample treatment, and the individual PFAS concentrations were corrected using their corresponding surrogates. Considering the significance of quantifying 6:2 Cl-PFESA and 6:2 H-PFESA in this study, the impact of matrix effects on PFESAs were validated. Known amounts of 6:2 Cl-PFESA and 6:2 H-PFESA (0.1 ng/mL and 1 ng/mL, respectively) were spiked into extraction solutions from sediment collected from the deep sea of the Western Pacific at depths >10 cm, which did not contain any PFESA before spiking (*n* = 3). The measured concentrations were compared to the theoretical values, showing deviations of less than 20% when ^13^C_8_–PFOS was used as the internal standard, confirming the reliability of using ^13^C_8_–PFOS for quantifying 6:2 Cl-PFESA and 6:2 H-PFESA.

### 2.5 Statistical Analysis

A nonparametric Mann–Whitney test was used to examine the significant difference. Spearman’s test was used for the correlation analysis using a two-tailed test. Statistical analysis was performed using IBM SPSS software (version 22.0, IBM Corp., NY, USA) and the R programming language. Statistical significance was accepted at *p* < 0.05 to examine significant differences.

### 2.6 Environmental Risk Assessment

Risk quotients (RQ) of individual PFAS were obtained via dividing the measured environmental concentration (MEC) obtained in this study by the environmental quality standard (EQS) or predicted no-effect concentration (PNEC) (i.e., RQ = MEC/EQS or RQ = MEC/PNEC). Due to limited ecotoxicological data on PFAS in sediments, an equilibrium partitioning method was applied using the seawater EQS (or PNEC) of PFAS in seawater and the organic carbon–water partition coefficient (*K*_oc_, cm^3^/g) of PFAS between sediment and bottom water calculated in this study and previous studies.^25,33–35^ Detailed information on the derivation of EQS, PNEC, and *K*_oc_ is provided in Text S4. Risk classification was based on the risk ranking criteria where RQ < 0.01 indicating “unlikely to pose risk”, 0.01 < RQ < 0.1 indicating “low risk”, 0.1 < RQ < 1 indicating “medium risk”, and RQ > 1 indicating “high risk”.^24^

## 3 Results and Discussion

### 3.1 PFAS in Surface Sediments

#### 3.1.1 Sediment from River Outlets

Thirty PFAS were detected in surface sediment samples collected from the river outlets, with the total PFAS concentrations (∑_30_ PFAS) ranging from 244 ± 39 pg/g dw (O2) to 14400 ± 2080 pg/g dw (O7) with an average of 4010 ± 4210 pg/g dw (Figure 2A). PFOS was the predominant compound, accounting for 18%–55% of ∑_30_ PFAS, followed by PFOA (4%–19%), 6:2 Cl-PFESA (1%–27%), and perfluoroundecanoate (PFUnDA, 0%–22%). Short-chain perfluoroalkyl carboxylates (S-PFCAs, perfluorocarbon chain length ≤ 6) and short-chain perfluoroalkyl sulfonates (S-PFSAs, perfluorocarbon chain length ≤ 5) in total contributed to less than 8% of ∑_30_ PFAS, while long-chain PFCAs (L-PFCAs, perfluorocarbon chain length ≥ 7) and long-chain PFSAs (L-PFSAs, perfluorocarbon chain length ≥ 6) accounted for 63%–86% of ∑_30_ PFAS in the investigated outlet sediment samples (Figure 2B).^17^

**Figure 2:**
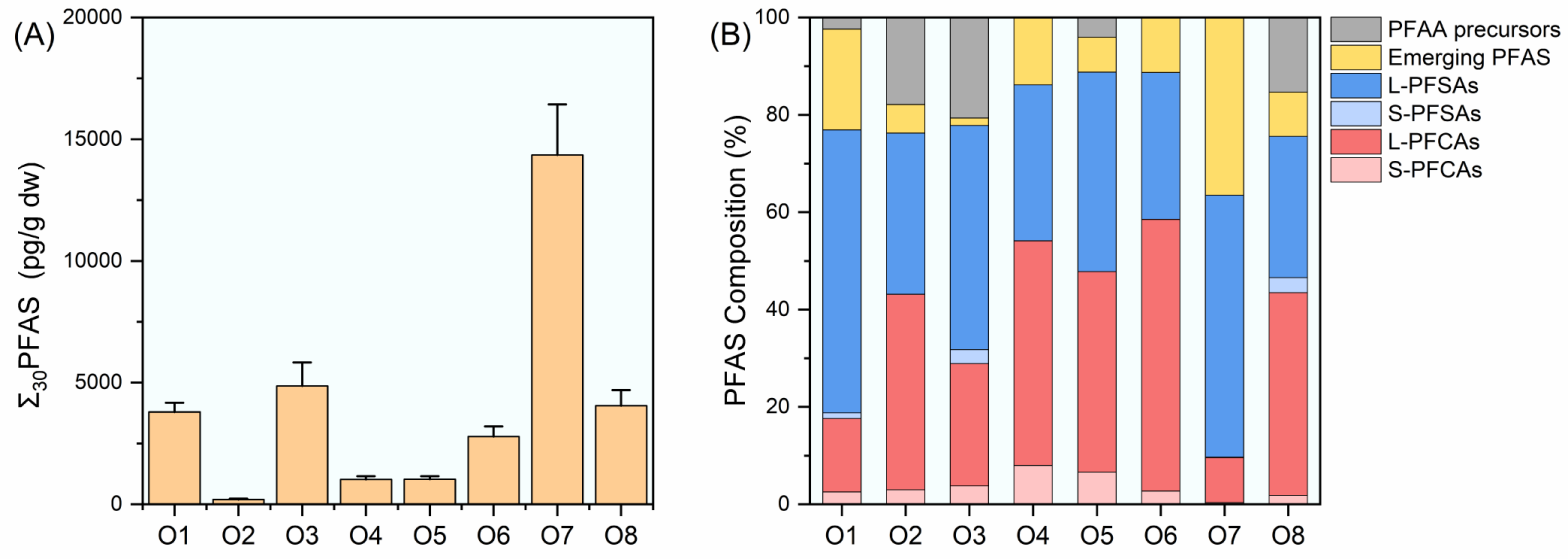
Mean (+SD) concentrations (A) and composition (B) of PFAS in surface sediment samples collected from the eight major Pearl River outlets.

Emerging PFAS were detected in all the outlet sediment samples and accounted for 2%–36% of ∑_30_ PFAS, revealing the ubiquitous distribution of emerging PFAS in the GBA, suggesting that their potential ecological risk could not be underestimated. Cl-PFESA was the predominant emerging PFAS group, with concentrations ranging from 9.34 to 4440 pg/g dw. The levels of many emerging PFAS, including PFECHS (<2.5–106 pg/g dw), 6:2 H-PFESA (<2.5–560 pg/g dw), 8:2 H-PFESA (<5–63.1 pg/g dw), and OBS (<5–51.3 pg/g dw), were reported for the first time in sediment of the PRE. In particular, two PFAAs, 6:8 and 8:8 perfluoroalkyl phosphinates (PFPiAs), were detected in O8 with concentrations of 15.4 and 6.93 pg/g dw, respectively. PFPiAs are used as surfactants in industrial and commercial applications while their occurrence in China has been less reported; this study is the first report on their occurrence in the PRE, implying their production and/or use in China.^36^ For PFAA precursors/intermediates, fluorotelomer phosphate diester was the predominant group, detected in six outlets with concentrations ranging from 14.2 to 1000 pg/g dw. *N*-ethyl perfluorooctane sulfonamido acetate (*N*-ethyl FOSAA) and *N*-methyl FOSAA were found in O8 (Humen, 276 pg/g dw) and O1 (Yamen, 63.8 pg/g dw) but not in other outlets. The widespread detection of multiple PFAS precursors highlights their significant contribution to PFAS in the sediment environment of the GBA. Spatially, the highest PFAS level was observed in sediment from O7 (Jiaomen, 14400 pg/g dw), predominated by PFOS (7370 pg/g dw) and 6:2 Cl-PFESA (3900 pg/g dw). Thirty PFAS were detected in O8, representing the highest diversity of PFAS inventory among the investigated river outlets. O7 and O8 are spatially close to each other (Figure 1), the significant urbanization and the presence of densely populated industrial parks (such as those involved in metal-plating and textiles) near the upstream of Jiaomen (O7) and Humen (O8) can contribute to the complex and elevated levels of PFAS pollution in these two river outlets.^24,29^

#### 3.1.2 Sediment from the NSCS

In surface sediment samples collected from the NSCS, 22 PFAS were detected, including 13 PFAAs, 5 emerging PFAS, and 4 PFAA precursors/intermediates, with the total PFAS concentrations (∑_22_ PFAS) ranged from 31.6 to 363 pg/g dw (mean ± SD: 160 ± 84.9 pg/g dw) (Figure 3A), which were much lower than those in the sediments collected from the river outlets (4020 ± 4200 pg/g dw). Similar PFAS compositions were observed between the marine sediments and the outlet sediments. PFOS, PFOA, PFUnDA, and 6:2 Cl-PFESA were the four predominant PFAS, which on average, contributed to 26%, 22%, 12%, and 5% of ∑_22_ PFAS, respectively. Regarding PFAAs, L-PFCAs was the predominant class, which accounted for 46% of ∑_22_ PFAS, followed by L-PFSAs (28%) and emerging PFAS (11%, Figure 3B). Spearman correlation was conducted for 11 PFAS, which had detection frequencies of over 50% across all sediment samples (Table S6). A significant positive correlation was observed among PFAS with perfluorocarbon chain lengths of 7–11 (*p* < 0.05), suggesting that these analogs in the NSCS sediments probably originated from similar sources. Another possible reason can be the origin of these PFAS from the transformation of the same PFAS precursors. The concentrations of 6:2 Cl-PFESA were significantly positively correlated with PFOS (*r* = 0.789, *p* < 0.05), implying a proximate origin between PFOS and this alternative with different functional groups. Spatially, PFAS concentrations were observed to decrease from the estuaries to the offshore regions, mainly due to the dilution effect (Figure S2). The decreasing trend for L-PFSAs and emerging PFAS was more evident than that for L-PFCAs. One possible explanation is a shift in their sources; for example, differences in PFAS composition and discharge volumes among various river outlets may play a role. Another explanation can be the distinct physicochemical properties of different PFAS, which influence their environmental behavior and distribution patterns. Another possible explanation is that PFAS with a sulfonic headgroup exhibit stronger associations with solids compared to those with a carboxylic group of the same carbon chain length. This results in a lower migration potential for PFSAs compared to PFCAs of the same carbon chain length.,^17,37,38^

**Figure 3:**
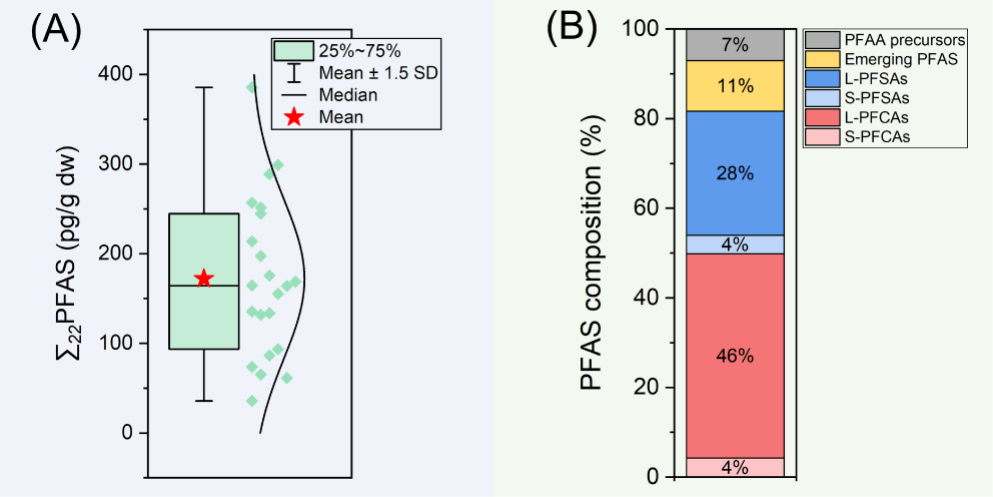
(A) Concentrations (pg/g dw) and (B) composition of PFAS in surface sediment samples collected from the NSCS.

### 3.2 Legacy PFAS in Sediment Cores

The dating results derived from the CIC model indicate that cores LD and DWB were deposited in a relatively stable manner, whereas cores TLH1C and DB2C experienced some degree of mixing during deposition (Figure S1). Consequently, the estimated deposition years for cores TLH1C and DB2C are less reliable. Therefore, in the subsequent discussion, cores LD and DWB are analyzed concerning their deposition years, while cores TLH1C and DB2C are discussed in relation to their depth.^39^

PFOS was first detected in core LD dated ∼1958 (8190 pg/g OC), and its levels increased to 21100 pg/g OC in ∼2013, then dropped to 4230 pg/g OC in ∼2017 (Figure 4A). Similarly, PFOS in core DWB was first detected in a layer dated ∼1971 (1540 pg/g OC), and its concentrations increased to 9290 pg/g OC in ∼2016; then they decreased to 4670 and 3110 pg/g OC in ∼2020 and ∼2021, respectively (Figure 4B). A trend of first increasing and then decreasing was also observed for PFOS in core DB2C with increasing depth (Figure S3). China is the world’s largest developing country. With regulatory restrictions on PFOS imposed in developed countries since the 2000s, China scaled up PFOS production to fill market gaps in the early days.^39,40^ In 2014, PFOS started to be restricted in China, while controls on PFOS have become more stringent since 2019. After many exemptions for PFOS expired, PFOS was completely banned on January 1, 2024.^41,42^ The temporal trend of first increasing and then decreasing PFOS concentrations in the investigated sediment core samples reflects the effectiveness of control measures on PFOS in China.

**Figure 4:**
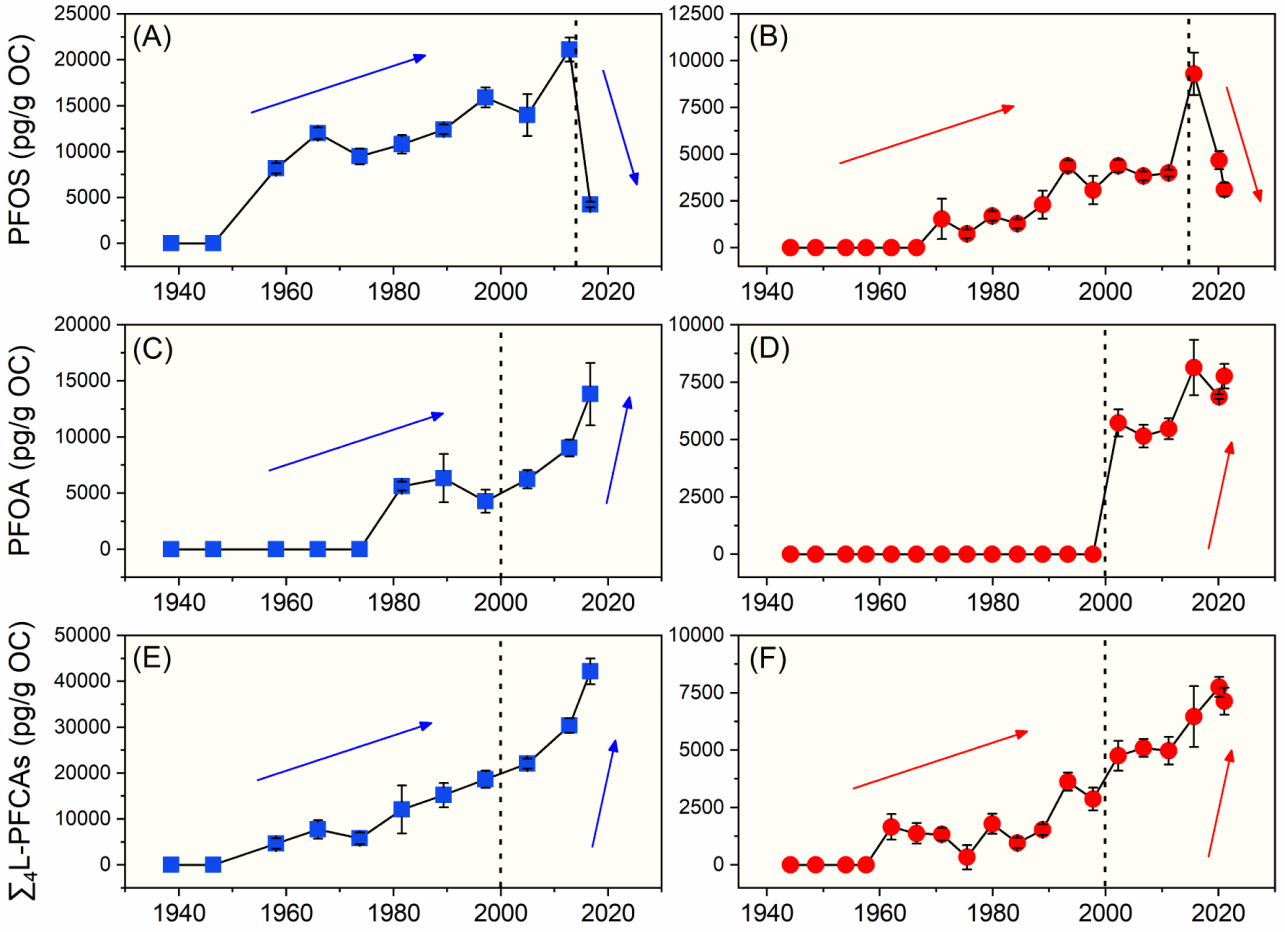
Temporal trend of the concentrations (mean ± SD) of PFOS (A and B), PFOA (C and D), and ∑_4_ L-PFCAs (perfluorocarbon chain length: 8–11, E and F) in sediment cores LD (left, blue points) and DWB (right, red points).

PFOA was first detected in core LD in ∼1982, and its levels remained relatively stable until ∼2005, with concentrations ranging from 4280 to 6330 pg/g OC; however, its levels reached 9030 and 13800 pg/g OC in ∼2013 and ∼2017, respectively (Figure 4C). In core DWB, PFOA was not detected until ∼ 2002 (5730 pg/g dw), and an increasing temporal trend was found (Figure 4D). In sediment cores DB2C and TLH1C, PFOA concentrations generally decreased with increasing depth, with the highest concentrations (80.6 ng/g dw for core DB2C and 380 ng/g dw for core TLH1C) observed in the surface layers of both sediment cores (Figure S3). In pace with the phasing out of PFOA in developed countries, China has become the largest PFOA manufacturer and user since 2002, and PFOA was not banned in China until March 1, 2023 (currently still with specific exemptions).^43,44^ Continuous production and use and lack of control are the main reasons for the increasing levels of PFOA in sediment core samples over the past two decades. In addition, the production and use of PFOA precursors may be a contributing factor, as these compounds can be eventually transformed into PFOA in the environment.^45^ Long-term follow-up monitoring of PFOA is a requisite to evaluate the effectiveness of its phase-out regulations in China, and more research on PFAS precursors will be a requisite.

Four L-PFCAs (i.e., PFNA, PFDA, PFUuDA, and PFDoDA) were widely detected in sediment core samples, and there was a clear temporal trend of increasing concentrations in both investigated cores. The total concentrations of these four L-PFCAs (with perfluorocarbon chain lengths of 8–11) (∑_4_ L-PFCAs) in cores LD and DWB increased from 4660 pg/g OC (in 1958) to 2790 pg/g OC (in ∼2017) and from 1660 pg/g OC (in ∼1962) to 7130 pg/g OC (in ∼2021), respectively (Figure 4E,F). In sediment cores DB2C and TLH1C, ∑_4_ L-PFCAs also generally decreased with increasing depth, with the highest concentrations (67.8 ng/g dw for core DB2C and 152 ng/g dw for core TLH1C) observed in the surface layers of both sediment cores (Figure S3). Currently, the production information on L-PFCAs in China remains unclear. The increasing levels of L-PFCAs observed in this study suggest that the production and/or use of L-PFCAs, particularly their precursors, in the GBA may have gradually risen over time. China currently has no regulatory policy for L-PFCAs. Note that L-PFCAs with perfluorocarbon chain lengths of 8–20 have been proposed for listing under the Stockholm Convention since 2022,^46^ and it is anticipated that China will incorporate L-PFCAs into its regulatory framework in the foreseeable future. Considering the increasing temporal trend of the concentrations of L-PFCAs in sediment cores, future efforts on the long-term monitoring of L-PFCAs will be critical.

Many studies have documented the concentrations of legacy PFAS (i.e., PFOS, PFOA, and L-PFCAs) in sediment cores collected from North America and Europe.^47–50^ The levels of legacy PFAS were found to be peaked in the mid-2000s and subsequently displayed a declining trend in the following decade. These findings are consistent with the historical production patterns of PFAS in these developed countries where the phasing out of PFAS commenced in the early 2000s. In the present work, levels of these legacy PFAS displayed a notable escalation after the mid-2000s in sediment cores collected in the PRE, which is adjacent to the GBA, one of the most developed regions in China. These results reflect the shift of the PFAS industry to China during the early 2000s. Furthermore, PFAA precursors and intermediates were rarely detected in our sediment core samples, likely due to their transformation in sediment. This may have influenced, to some extent, the temporal trends observed in PFAA concentrations in the studied sediment cores.

### 3.3 Emerging PFAS in Sediment Cores

Six emerging PFAS were detected in sediment core samples, including 6:2 and 8:2 Cl-PFESAs, 6:2 and 8:2 H-PFESAs, PFECHS, and OBS.

#### 3.3.1 PFECHS and OBS

PFECHS was detected in cores DWB and LD in ∼2002 and ∼2005, respectively, and its concentrations exhibited an increasing temporal trend ever since (Figure S4). The primary usage of PFECHS is as an additive in hydraulic fluid in aircraft, which has been used for over 70 years.^9,11^ Nevertheless, PFECHS was not detected in our sediment core slices earlier than 2000, and the time point when PFECHS was first detected (i.e., ∼2002 and ∼2005) was in line with the large-scale production and use of PFOS in China (i.e., the early 2000s). This result implies the recent application of PFECHS in the GBA over the past decade. There exists only one previous study on the investigation of the PFECHS temporal trend in Arctic Lake sediment cores, in which, however, PFECHS was not found in those samples.^51^ The present study is the first record on the historical pollution status of PFECHS in sediment cores. In addition, PFECHS was in all layers down to a depth of 81 cm in core TLH1C, indicating the releases of PFECHS in the studied area in recent years (Figure S5). Regarding surface sediment samples from the NSCS, the highest concentrations of PFECHS were found at sites P1 (14.2 pg/g dw) and P7 (15.7 pg/g dw), which are closest to the Hong Kong and Shenzhen airports, respectively. A significant positive correlation was observed in concentrations between PFOS and PFECHS in both the surface sediment and sediment core samples (*n* = 16, *r*_*s*_ = 0.509, *p* < 0.05). Collectively, our results suggest that in the GBA, the production and use associated with PFOS, especially emission from the airport (e.g., aircraft hydraulic fluids), should be the primary PFECHS contributor. Generally, PFECHS concentrations in sediments were relatively low (surface sediment: < 106 pg/g dw; sediment core: < 13.0 pg/g dw). Our previous studies have evidenced the increasing pollution levels in stranded porpoises and biomagnification potential of PFECHS in an estuarine food web;^25,26^ combined with the present results about the increasing trend of PFECHS levels in sediment cores, studies on its deposition in sediments should not be omitted from future work.^25^

OBS was detected in slices of core DWB with estimated years of ∼2011 (8.61 pg/g dw) and ∼2016 (20.0 pg/g dw) but not detected in the other slices of DWB, neither in cores LD, DB, and TLH1C. Similarly, OBS was less detected and at low levels in surface sediments from the river outlets (4 out of 8, < 51.3 pg/g dw) and the NSCS (3 out of 22, < 9.98 pg/g dw). OBS-related products, with the trade name Neos Ftergent, have been produced by a Japanese company since the 1980s. However, information regarding the production and use of OBS in China remains insufficient. The rare detection of OBS in the investigated sediment core samples precludes temporal trend evaluation. Still, the low levels of OBS in the surface sediments indicate that OBS pollution is relatively inconsiderable in the PRE.

#### 3.3.2 Cl-PFESA and H-PFESAs: Ratios

As the major component of F-53B, 6:2 Cl-PFESA has been synthesized since the 1970s, which is applied as a mist suppressant in the electroplating industry in China only.^8^ H-PFESAs are the impurity in the product F-53B, and the mass ratio of 6:2 H-PFESA to 6:2 Cl-PFESA in commercial F-53B is less than 2%.^12^ Nevertheless, this ratio is much greater in the natural aquatic environment as it is reported to be 1.3%–280% (mean = 27%) and 4%–68% (mean = 19%) in the freshwater and coastal water, respectively.^25,28^ These results suggest that 6:2 H-PFESA in the aquatic environment does not originate from F-53B alone, and the reductive dechlorination of 6:2 Cl-PFESA shall be an important contributor.^12,52^ However, the occurrence of H-PFESAs in sediments is less reported, and related studies are mostly based on nontarget screening identification, making the environmental fate of H-PFESAs remains unclear due to the uncertainty brought by semiquantification using HRMS.^26,53^

In the surface sediment collected near metal-plating facilities where F-53B was used, the levels of Cl-PFESAs were reported about an order of magnitude higher than those of H-PFESAs (i.e., the ratio of 6:2 H-PFESA to 6:2 Cl-PFESA ≈ 0.1).^12^ However, in the present study, the ratios of 6:2 H-PFESA to 6:2 Cl-PFESA in surface sediment samples collected from the Pearl River outlets and the NSCS ranged from 0.144 to 1.10 and from 0.151 to 1.39, with means of 0.365 ± 0.316 and 0.465 ± 0.309, respectively. This result implies the contribution of 6:2 Cl-PFESA reductive dechlorination as an essential source for 6:2 H-PFESA in sediments. Notably, the ratios of 6:2 H-PFESA to 6:2 Cl-PFESA in the four investigated sediment cores ranged from 1.63 to 11.2 with a mean of 5.36 ± 2.74, which were significantly higher than those in surface sediment samples collected from the Pearl River outlets and the NSCS (Figure 5A). In addition, a significant negative correlation was observed between the ratios of 6:2 H-PFESA to 6:2 Cl-PFESA and the estimated deposition year in cores LD and DWB (Spearman correlation, *p* < 0.05, *n* = 13; Figure 5B). These results evidence the transformation of 6:2 Cl-PFESA to 6:2 H-PFESA in the sediment over time, and H-PFESAs should be integrated into evaluating the pollution status and the ecological risk of Cl-PFESAs. Regarding 8:2 Cl-PFESA and 8:2 H-PFESA, they were less detected in the investigated sediment samples, limiting the discussion on their ratios. In the surface sediment samples, the detection frequency of 8:2 Cl-PFESA (17%) was higher than that of 8:2 H-PFESA (10%). However, an opposite result was observed in the sediment core samples where the detection frequency of 8:2 Cl-PFESA was 3%, which was much lower than that of 8:2 H-PFESA (30%). This result implies the transformation of 8:2 Cl-PFESA to 8:2 H-PFESA may occur in sediments.

**Figure 5:**
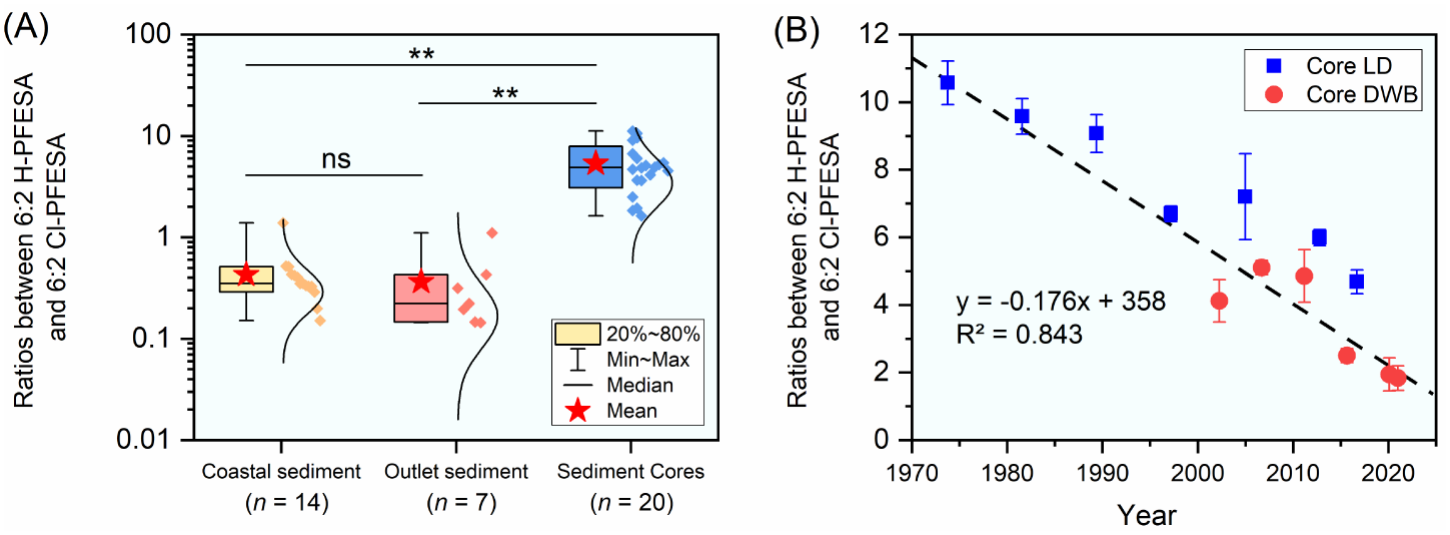
Ratios between 6:2 H-PFESA and 6:2 Cl-PFESA in (A) different types of sediment samples and (B) sediment cores LD and DWB. Data are presented as mean ± SD; ns represents the difference is not significant and ** represents *p* < 0.01).

#### 3.3.3 Cl-PFESAs and H-PFESAs: Temporal Trend

As 8:2 Cl-PFESA and 8:2 H-PFESA were much less detected in the sediment core samples, the following discussion regarding the temporal trend of PFESAs focuses on 6:2 Cl-PFESA and 6:2 H-PFESA. 6:2 Cl-PFESA was first detected in cores LD and DWB with estimated years of ∼1974 and ∼2002, respectively (Figure 6A,B). The time when 6:2 Cl-PFESA was first detected in core LD aligns with its application in China (i.e., in the 1970s).^8^ A general increasing temporal trend was observed for 6:2 Cl-PFESA concentrations in both cores LD and DWB. Despite the fact that the production and usage data of F-53B is not clear, it is expected that F-53B will have a higher market valuation in China following a global move to phase out PFOS, which is supposed to lead to the increasing trend of 6:2 Cl-PFESA in recent years. To the best of our knowledge, only two studies have investigated the temporal trends of Cl-PFESAs in abiotic samples.^51,54^ However, due to their infrequent detection (detection frequencies <10%), data on the temporal trends of Cl-PFESAs in abiotic samples are lacking. The present study provides the first report on the historical pollution dynamics of Cl-PFESAs in the environment. There exist some studies on the temporal trend of Cl-PFESAs in biotic samples collected in the past two decades. In liver samples collected from stranded finless porpoises and Indo-Pacific humpback dolphins from the NSCS between 2012 and 2018, no significant temporal trend was found for 6:2 Cl-PFESA concentrations, but a significant decreasing trend was observed for the levels of 8:2 Cl-PFESA.^26^ In the cord plasma collected from the Chinese general population, the levels of 6:2 and 8:2 Cl-PFESAs exhibited a decreasing trend from 2003 to 2018.^55^ Interestingly, the increasing trend of 6:2 Cl-PFESA in our sediment core samples was inconsistent with those previously reported in biotic samples. The main reason, as stated before, is likely attributed to the transformation of Cl-PFESAs to H-PFESAs in the sediment environment, which led to lower levels of 6:2 Cl-PFESA in sediment slices from earlier years.

**Figure 6:**
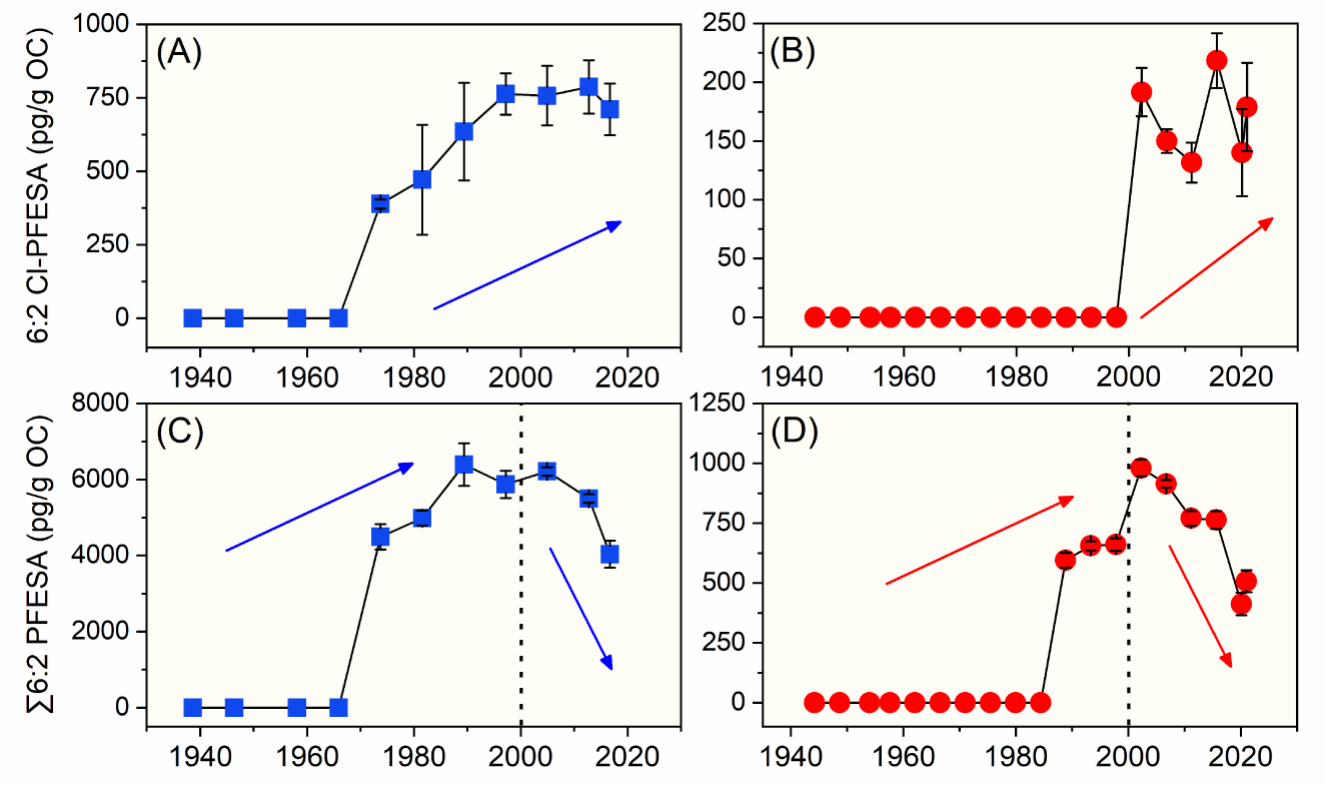
Temporal trend of the concentrations of 6:2 Cl-PFESA and ∑6:2 PFESAs in sediment cores LD (left, blue points) and DWB (right, red points).

When taking 6:2 Cl-PFESA and 6:2 H-PFESA together, ∑6:2 PFESAs (i.e., the sum concentration of 6:2 Cl-PFESA and 6:2 H-PFESA) exhibited a decreasing trend after 2000 (Figure 6C,D). F-53B was the only available mist suppressant in the Chinese electroplating industry until the use of FC-80 (C_8_F_17_O_3_SK) and FC-248 (C_16_H_20_F_17_O_3_NS) in the 1980s.^8^ However, a recent investigation on PFAS in representative chrome mist suppressants, collected from the Chinese market over the past 10 years, evidence that PFOS, rather than Cl-PFESAs, was the main component.^56^ Our result implies that the production and use of Cl-PFESAs in China decreased after 2000, which may result from the increasing production and use of PFOS in China after its phasing out in developed countries. PFOS has been restricted in China since 2014 but exempted its use in the electroplating industry until 2019.^41,42^ Therefore, no increasing trend was observed for levels of ∑6:2 PFESAs in the investigated sediment cores. Nevertheless, the continuous monitoring of PFESAs, important substitutes for PFOS, is essential given the complete ban on PFOS since 2024 in China.^44^

### 3.4 Historical Ecological Risk Assessment of PFAS

The historical variations in the ecological risk of PFAS were examined through the levels of PFAS in sediment cores. RQs were calculated for four predominant PFAS in the sediments, including PFOS, PFOA, PFUnDA, and 6:2 Cl-PFESA. These four PFAS collectively accounted for 73% and 65% of the total PFAS concentrations in surface sediments from the river outlets (∑_30_ PFAS) and the NSCS (∑_22_ PFAS), respectively. The EQSs of PFOS and PFOA followed the values set by the European Union and Italian Parliament, which are 0.13 ng/L and 20 ng/L in saltwater for PFOS and PFOA, respectively.^57,58^ However, the EQSs of PFUnDA and 6:2 Cl-PFESA are lacking. For PFUnDA, a PNEC of 436 ng/L was applied based on its developmental toxicity to zebrafish.^59^ The PNEC of 6:2 Cl-PFESA was set as 0.13 ng/L to protect marine top predators, and details regarding the derivation of PENCs can be found in Text S4. The *K*_oc_ of individual PFAS was calculated based on their levels in the sediment (as determined in this study) and the bottom seawater at the same sites (as determined in our previous work).^25^ The field-based logarithmic *K*_oc_ were 3.70 ± 0.48, 4.98 ± 0.13, 4.38 ± 0.41, and 4.34 ± 0.48 for PFOS, PFOA, PFUnDA, and 6:2 Cl-PFESA, respectively. The resultant RQs of PFUnDA were found to be less than 0.01 in all the sediment samples, indicating that PFUnDA was unlikely to pose a risk to benthic organisms in the PRE. The RQs for the other three PFAS in the sediment cores are presented in Figure 7.

**Figure 7:**
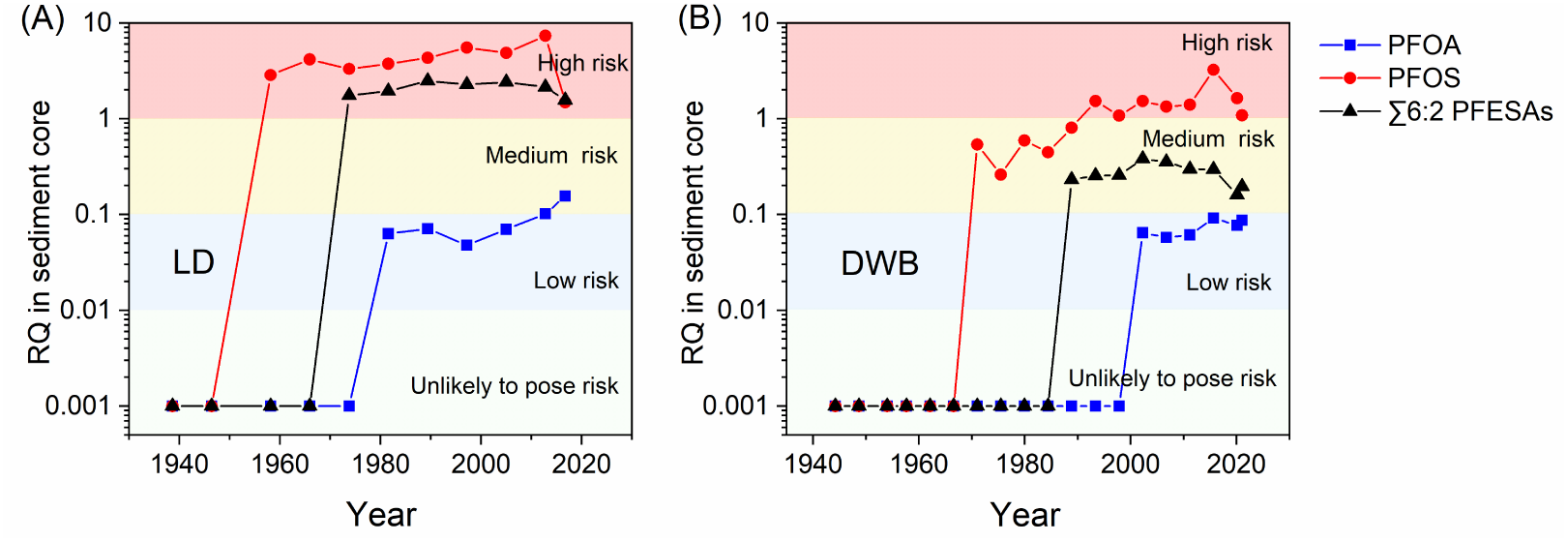
RQs of PFOA, PFOS, and ∑6:2 PFESAs in sediment cores (A) LD and (B) DWB.

Regarding the RQs of PFOA, despite an increasing trend in recent years, the values generally remained below 0.1 in both cores LD and DWB, indicating a low ecological risk associated with PFOA in the studied region. With the ban on PFOA implemented in China in 2023, the ecological risk linked to PFOA is anticipated to remain low.^60^ In contrast, PFOS has consistently posed a high ecological risk (i.e., RQ > 1.0) since the 1960s in core LD and the 1990s in core DWB. However, after the 2010s, the RQs of PFOS gradually declined, indicating a shift to medium risk. This trend underscores the effectiveness of regulatory measures implemented in China.

The structural similarity between 6:2 H-PFESA and 6:2 Cl-PFESA suggests the potential for comparable toxic effects between these substances; however, experimental toxicological data for 6:2 H-PFESA remain unavailable. The ECOSAR predictive model was utilized to evaluate differences in aquatic toxicity between 6:2 Cl-PFESA and 6:2 H-PFESA (Table S7).^60^ The estimated median effective concentration (EC_50_), median lethal concentration (LC_50_), and no-observed-effect concentration (NOEC) of 6:2 H-PFESA were higher than those of 6:2 Cl-PFESA, although they were within the same order of magnitude. These results suggest that 6:2 H-PFESA likely exhibits lower toxicity compared to 6:2 Cl-PFESA. On the other hand, as previously discussed, 6:2 Cl-PFESA undergoes dechlorination in sediment, gradually converting into 6:2 H-PFESA. Therefore, to better evaluate the potential ecological risks associated with both 6:2 Cl-PFESA and 6:2 H-PFESA, their concentrations were combined and compared against the risk assessment threshold of 6:2 Cl-PFESA in the present study. The ecological risks associated with ∑6:2 PFESAs remained relatively stable over time, and ∑6:2 PFESAs were identified as posing a high risk since 1973 (RQs: 1.56 to 2.47) and a medium risk since 1989 (RQs: 0.159 to 0.379) in cores LD and DWB, respectively. These findings underscore the noteworthy ecological risk posed by 6:2 PFESAs in the PRE and the NSCS, emphasizing the importance of further research on the ecological impacts of Cl-PFESAs by taking H-PFESAs into account within the global framework of PFOS phase-out.

## 4 Environmental Implications

Marine sediment is recognized as a significant reservoir of PFAS in the environment. The wide detection of emerging PFAS in the sediments from the river outlets and adjacent sea underscored the importance of considering contamination from these emerging PFAS. The sediment core data indicate a decline in PFOS levels in recent years, likely linked to regulatory measures on PFOS implemented in China in 2014. However, pinpointing the exact year of trend change remains challenging due to uncertainties in the sediment core dating process. PFOA and L-PFCAs with perfluorocarbon chain lengths of 8–11 showed an increasing trend from the 1980s to the 2020s, which can be attributed to the absence of regulations on these PFAS in China. Notably, levels of PFOA and L-PFCAs exhibited an accelerating increasing trend since the mid-2000s, aligning with the timeline of legacy PFAS bans in developed countries. This result reflects the fact that the PFAS industry shifted to China in the early 2000s.

This study, for the first time, presents the temporal variation trend of PFECHS and PFESAs in environmental media. Although PFECHS occurred at relatively low concentration levels (<12.2 pg/g dw), future investigation on PFECHS is necessary due to its increasing temporal pollution trend and significant biomagnification potential. The study reveals the widespread presence of H-PFESAs, an understudied class of emerging PFAS, in the sediment samples. The concentration ratios of 6:2 H-PFESA to 6:2 Cl-PFESA in the sediment cores were significantly higher than those in estuarine and nearshore surface sediments, and such ratios showed an increasing trend as the sediment ages increased. H-PFESAs are considered stable degradation products of Cl-PFESAs, implying their significance as a final form of Cl-PFESAs in the environment. Focusing on investigating Cl-PFESAs only without considering H-PFESAs may lead to underestimated ecological risks and inaccurate estimation of concentration variation trends. Therefore, further field investigations and toxicity studies on H-PFESAs are warranted. In addition, considering the widespread detection of H-PFAAs in the environment, our results suggest that the transformation of PFAA precursors (e.g., Cl-PFAAs) may serve as a significant source. This finding highlights the probability of latent and unrecognized risks associated with these PFAS, which might have been underestimated. Moreover, the scale of production and use of Cl-PFESAs are currently unclear. By summing up the concentrations of 6:2 H-PFESA and 6:2 Cl-PFESA, the concentration of ∑6:2 PFESAs has declined from 2000 to the present time, despite the absence of regulatory measures for Cl-PFESAs. This trend may be associated with the extensive application of PFOS as chrome mist suppressants in the Chinese market since the early 2000s.^61^ However, continuous attention on PFESAs is needed within the context of completely eliminating PFOS in China.

A preliminary environmental risk assessment indicates that PFOA and PFUnDA may not bring significant ecological risk in the PRE. However, Cl-PFESAs and PFOS presented medium to high risks. Over the past decade, the RQs of PFOS have shown a gradual decrease, suggesting the effectiveness of PFOS restrictions implemented in China. Nevertheless, the ecological risks associated with Cl-PFESAs have remained relatively stable in the past 40 years, underscoring the importance of establishing control measures for these PFOS alternatives in the context of the phase-out of PFOS globally.

## Supplementary Material



## Notes

Details on standards and reagents, sample treatment, and instrumental analysis; sediment dating method and results; TOC content; spearman correlation results; toxicity data predicted by ECOSAR model; matrix-spiked MQLs and recoveries; spatial distribution of PFAS in surface sediment samples; and PFAS concentrations in sediment cores DB2C and TLH1C (PDF)
