# Supplementary material for: Spatiotemporal Trend of PFAS in Estuarine Sediments: Insights into Chlorinated Polyfluoroalkyl Ether Sulfonate Transformation
Source: Environ Sci Technol. 2025 Apr 2;59(14):7377–88. doi: 10.1021/acs.est.5c02731 (PMC12713727; doi:10.1021/acs.est.5c02731)
Supplement: Supplementary file 1 [file es5c02731_si_001.pdf]

Supporting Information (SI)

Spatiotemporal trend of PFAS in estuarine sediments: Insights into chlorinated polyfluoroalkyl ether sulfonate transformation

Qi Wang [a,b,c], Yuefei Ruan [a,b,c,d] *, Yetong Shao [a,b], Linjie Jin [a,b], Naiyu Xie [a], Xiaoqiang Yang [c], Yuanyuan Hong [e], He Wang [e], Akira Tsujimoto [f], Moriaki Yasuhara [a,e], Kenneth M. Y. Leung [a,c,d,g], Paul K. S. Lam [a,c,d,h]

[a] *State Key Laboratory of Marine Pollution (SKLMP), City University of Hong Kong, Hong Kong SAR 999077, China*

[b] *Research Centre for the Oceans and Human Health, City University of Hong Kong Shenzhen Research Institute, Shenzhen 518057, China*

[c] *Southern Marine Science and Engineering Guangdong Laboratory (Zhuhai), Zhuhai 519080, China*

[d] *Department of Chemistry, City University of Hong Kong, Hong Kong SAR 999077, China*

[e] *School of Biological Sciences, Area of Ecology and Biodiversity, Swire Institute of Marine Science, Institute for Climate and Carbon Neutrality, and Musketeers Foundation Institute of Data Science, The University of Hong Kong, Hong Kong SAR 999077, China*

[f] *Institute of Education, Academic Assembly, Shimane University, Shimane 690-8504, Japan*

[g] *School of Energy and Environment, City University of Hong Kong, Hong Kong SAR 999077, China*

[h] *Department of Applied Science, School of Science and Technology, Hong Kong Metropolitan University, Hong Kong SAR 999077, China*

This document contains 17 pages, 4 texts, 7 tables, and 5 figures.

Table of Contents

Page

Texts

Text S1. Details on the standards S3

Text S2. Details on the sediment dating S3

Text S3. Details on sample treatment and quantification method S4

Text S4. Derivation of EQS, PNECs, and K_{oc} S5

Tables

Table S1. Chemical name, abbreviation, class, and formula of PFAS S7

Table S2. Depth and estimated deposition year in each sediment core layer S8

Table S3. TOC content (‰) in the sediment samples S8

Table S4. Mass spectrometer parameters for the analysis of PFAS S9

Table S5. Details on the recoveries and method quantification limits S11

Table S6. Spearman correlation results on PFAS in surface sediment samples collected from the northern South China Sea S12

Table S7. Toxicity data of 6:2 Cl-PFESA and 6:2 H-PFESA predicted by ECOSAR model S12

Figures

Figure S1. ln $^{210}Pb_{ex}$ in different slices of sediment cores S13

Figure S2. Spatial distribution of PFAS in surface sediment samples collected from the NSCS S13

Figure S3. Concentration of PFOS, PFOA, and $\sum_4$ L-PFCAs in sediment cores DB2C and TLH1C S14

Figure S4. Concentration of 6:2 Cl-PFESA and PFECHS in sediment cores LD and DWB S14

Figure S5. Concentration of 6:2 Cl-PFESA and PFECHS in sediment cores DB2C and TLH1C S15

References S16

Text S1. Details on the standards

Lithium bis(trifluoromethanesulfonyl)imide (NTf2 or LiTFSI, purity > 99%) was purchased from Sigma-Aldrich (Oakville, Canada), and OBS (purity > 94%) was purchased from Rhawn (Guangzhou, China). 6:2 and 8:2 H-PFESAs (purity > 95%) were donated by the Chinese Academy of Sciences (Beijing, China). The remaining 37 analytical standards and mass-labeled PFAS surrogates (purity > 97%) were purchased from Wellington Laboratories Inc. (Guelph, Canada).

Text S2. Details on the sediment dating

The sediment cores were dated using the ^{210}Pb excess and ^{137}Cs techniques. The ^{137}Cs was not detected in this work. For core DWB, γ-spectrometry was performed on sediment samples using a well-type, EGPC 150-P16 Ge-detector (Canberra-Eurysis), for 85,000 seconds.[1] Regarding core LD, a GWL-120-15 Ge-detector (AMETEK-AMT ORTEC Co.) was used with a measure time of 40,000 to 80,000 seconds. As the measured value of ^{210}Pb is composed of both supported and unsupported ^{210}Pb, the supported ^{210}Pb is ascribed to production from the parent ^{226}Ra in sediment. Therefore, the supported ^{210}Pb concentration, calculated from the γ-peaks of ^{214}Pb, was subtracted from the total ^{210}Pb concentration to obtain the unsupported ^{210}Pb concentration (i.e., ^{210}Pb$_{ex}$, hereafter).

The Constant Initial ^{210}Pb Concentration (CIC) model was applied to predict the deposition year of each sediment core slices:[1, 2]

$$C_i = C_0 e^{-\lambda t}$$

$$t = \frac{1}{\lambda} ln(\frac{C_0}{C_i})$$

where C_0 is the ^{210}Pb$_{ex}$ concentration in the surface sediment, and C_i is the concentration in the i-th core section. λ is the ^{210}Pb decay-constant, and t is the age of the sediment. The results can be found in Table S2 and Figure S1.

Text S3. Details on sample treatment and quantification method

Sediment samples were freeze-dried, homogenized, and weighed in polypropylene (PP) tubes. After 2 ng of mixed mass-labeled surrogates were added, 1.0-1.5 gram of homogenized freeze-dried sediment samples were sonicated with 5 mL of methanol at 25 ℃ for 30 min. Supernatants were collected after separated by centrifuge at 4000 g at room temperature for 5 min. The extraction by sonication was repeated twice, and the combined extracts (approx. 15 mL) were concentrated to approx. 2 mL under a gentle stream of high-purity nitrogen at 40 ℃ for extract cleanup. ENVI-Carb cartridges were preconditioned by passing through 2 mL of methanol three times. The concentrated extracts were loaded onto the preconditioned cartridges, and the eluates were collected. Analytes were further eluted via passing through 1 mL of methanol three times. The combined eluates were concentrated to 0.5 mL under a gentle stream of high-purity nitrogen at 40 ℃ and transferred to a PP chromatographic vial for instrumental analysis. Two ng of mixed mass-labeled surrogates were added to an empty PP tube and prepared along with each sample treatment as procedural blank. The PFAS recovery tests were conducted using sediment samples far offshore in the South China Sea with low PFAS concentrations (< 10 pg/g dw). Background PFAS concentrations were subtracted before calculating the recoveries. The recoveries of the surrogates fell within the range of 50%−120%

The instrumental method for the analysis of 45 target PFAS followed our previous studies.[3, 4] The determination of PFAS was performed using an Agilent 1290 Infinity ultra-performance liquid chromatograph (Agilent, Palo Alto, CA, USA) interfaced with a 5500 QTRAP® mass spectrometer in negative ion electrospray mode (Sciex, Foster City, CA, USA). All target PFAS were detected using multiple reaction monitoring. A gradient mobile phase of 2 mM ammonium acetate in Milli-Q® water/methanol 95:5 (solvent A) and 2 mM ammonium acetate in acetonitrile (solvent B) was used. At a flow rate of 0.3 mL/min, the gradient condition (in a sequence of 10% solvent B) was increased to 85% at 5 min, 100% at 9 min, and converted to original conditions, with a total run time of 15 min.

Text S4. Derivation of EQS, PNECs, and K_{oc}

RQs were calculated for four predominant PFAS in the sediments, including PFOS, PFOA, PFUnDA, and 6:2 Cl-PFESA. These four PFAS collectively accounted for 73% and 65% of the total PFAS concentrations in the surface sediments from river outlets and the coastal SCS, respectively.

The EQSs of PFOS and PFOA followed the values set by the European Union and Italian Parliament, which are 0.13 ng/L and 20 ng/L for PFOS and PFOA in saltwater, respectively.[5, 6] The EQS for PFUnDA and 6:2 Cl-PFESA in the environment are lacking. Significant morphological abnormality was observed in the zebrafish (AB strain) exposed to PFUnDA at a concentration of 4.36 mg/L. Hence, the PNEC value for PFUnDA in freshwater was set at 4.36 μg/L. The PNEC value of this compound in seawater was derived from the freshwater toxicological data with an extra assessment factor of 10.[7, 8] Therefore, the seawater PNEC value of PFUnDA used in this risk assessment was set at 436 ng/L. 6:2 Cl-PFESA exhibited notable biomagnification potential in the marine food web,[4, 9] which could exacerbate the ecological risk of 6:2 Cl-PFESA in top predators. In our previous study, the field-based bioaccumulation factors (BAFs, kg/L) of 6:2 Cl-PFESA were estimated to be 6.41 and 5.77 for two resident marine cetaceans Indo-Pacific humpback dolphin (*Sousa chinensis*) and finless porpoise (*Neophocaena phocaenoides*), respectively.[4] Using the tentative critical concentrations (TCCs) of 98.3 and 114.7 ng/g wet weight for 6:2 Cl-PFESA in dolphins and porpoises, respectively, for risk assessment evaluation,[10] the estimated PNECs of 6:2 Cl-PFESA in seawater were 0.038 and 0.19 ng/L for protecting dolphins and porpoises, respectively. These values are comparable to the EQS of PFOS set by EU (0.13 μg/L), consistent with the finding that 6:2 Cl-PFESA exhibits comparable toxicity to PFOS.[11] To lessen the uncertainty in the TCC risk assessment, the EQS of PFOS (i.e., 0.13 μg/L) was used to estimate the ecological risk of 6:2 Cl-PFESA in the present study.

The K_{oc} of individual PFAS was calculated based on their levels in the sediment (as determined in this study) and the bottom seawater at the same sites (as determined in our previous work).[4] Field-based K_{oc} was calculated to describe the partitioning of PFAS between water and sediment in the field. Water-sediment distribution coefficient (K_d, cm^3/g) was calculated according to the following equation:

$$K_d = \frac{C_S}{C_W} \times 1000$$

C_s is PFAS concentration in sediment (ng/g dw, as determined in this study) and C_w is PFAS concentration in the bottom seawater at the same sites (ng/L, as determined in our previous work).[4]

The organic carbon normalized distribution coefficient (K_{oc}, cm^3/g) of PFAS between sediment (SPM) and water dissolved phased was calculated as follows:

$$K_{oc} = \frac{K_d}{f_{oc}} \times 100$$

f_{oc} is the percentage of organic carbon in sediment (%). The calculated field-based logarithmic K_{oc} were 3.70 ± 0.48, 4.98 ± 0.13, 4.38 ± 0.41, and 4.34 ± 0.48 for PFOS, PFOA, PFUnDA, and 6:2 Cl-PFESA, respectively.

Table S1. Chemical name, abbreviation, class, and formula of each studied PFAS

Name	Abbreviation	Class	Formula
Perfluorobutanoate	PFBA	PFCA	$C_3F_7CO_2H$
Perfluoropentanoate	PFPeA	PFCA	$C_4F_9CO_2H$
Perfluorohexanoate	PFHxA	PFCA	$C_5F_{11}CO_2H$
Perfluoroheptanoate	PFHpA	PFCA	$C_6F_{13}CO_2H$
Perfluorooctanoate	PFOA	PFCA	$C_7F_{15}CO_2H$
Perfluorononanoate	PFNA	PFCA	$C_8F_{17}CO_2H$
Perfluorodecanoate	PFDA	PFCA	$C_9F_{19}CO_2H$
Perfluoroundecanoate	PFUnDA	PFCA	$C_{10}F_{21}CO_2H$
Perfluorododecanoate	PFDoDA	PFCA	$C_{11}F_{23}CO_2H$
Perfluorotetradecanoate	PFTeDA	PFCA	$C_{13}F_{27}CO_2H$
Perfluorohexadecanoate	PFHxDA	PFCA	$C_{15}F_{31}CO_2H$
Perfluorooctadecanoate	PFOcDA	PFCA	$C_{17}F_{35}CO_2H$
Perfluorobutanesulfonate	PFBS	PFSA	$C_4F_9SO_3H$
Perfluoropentanesulfonate	PFPeS	PFSA	$C_5F_{11}SO_3H$
Perfluorohexanesulfonate	PFHxS	PFSA	$C_6F_{13}SO_3H$
Perfluoroheptanesulfonate	PFHpS	PFSA	$C_7F_{15}SO_3H$
Perfluorooctanesulfonate	PFOS	PFSA	$C_8F_{17}SO_3H$
Perfluorononanesulfonate	PFNS	PFSA	$C_9F_{19}SO_3H$
Perfluorodecanesulfonate	PFDS	PFSA	$C_{10}F_{21}SO_3H$
2,3,3,3-Tetrafluoro-2-(heptafluoropropoxy)propanoate	HFPO-DA	Emerging PFAS	$C_6HF_{11}O_3$
6:2 Chlorinated polyfluorinated ether sulfonate	6:2 Cl-PFESA	Emerging PFAS	$C_8HClF_{16}O_4S$
8:2 Chlorinated polyfluorinated ether sulfonate	8:2 Cl-PFESA	Emerging PFAS	$C_{10}HClF_{20}O_4S$
4,8-Dioxa-3H-perfluorononanoate	ADONA	Emerging PFAS	$C_7H_2F_{12}O_4$
Perfluoroethylcyclohexane sulfonate	PFECHS	Emerging PFAS	$C_8HF_{15}O_3S$
Chlorinated perfluorooctanesulfonate	Cl-PFOS	Emerging PFAS	$C_8HClF_{16}O_3S$
6:2 Hydrogen-substituted polyfluorinated ether sulfonate	6:2 H-PFESA	Emerging PFAS	$C_8H_2F_{16}O_4S$
8:2 Hydrogen-substituted polyfluorinated ether sulfonate	8:2 H-PFESA	Emerging PFAS	$C_{10}H_2ClF_{20}O_4S$
p-Perfluorous nonenoxybenzene sulfonate	OBS	Emerging PFAS	$C_{15}H_5F_{17}O_4S$
Lithium bis(trifluoromethanesulfonyl)imide	NTf2	Emerging PFAS	$C_2F_6LiNO_4S_2$
4:2 Fluorotelomer sulfonate	4:2 FTSA	Precursor/intermediate	$C_6H_5F_9O_3S$
6:2 Fluorotelomer sulfonate	6:2 FTSA	Precursor/intermediate	$C_8H_5F_{13}O_3S$
8:2 Fluorotelomer sulfonate	8:2 FTSA	Precursor/intermediate	$C_{10}H_5F_{17}O_3S$
10:2 Fluorotelomer sulfonate	10:2 FTSA	Precursor/intermediate	$C_{12}H_5F_{21}O_3S$
3:3 Fluorotelomer carboxylate	FPrPA	Precursor/intermediate	$C_6H_5F_7O_2$
5:3 Fluorotelomer carboxylate	FPePA	Precursor/intermediate	$C_8H_5F_9O_2$
7:3 Fluorotelomer carboxylate	FHpPA	Precursor/intermediate	$C_{10}H_5F_{11}O_2$
6:2 Fluorotelomer phosphate diester	6:2-diPAP	Precursor/intermediate	$C_{16}H_9F_{26}O_4P$
6:2/8:2 Fluorotelomer phosphate diester	6:2/8:2-di PAP	Precursor/intermediate	$C_{18}H_9F_{30}O_4P$
8:2 Fluorotelomer phosphate diester	8:2 di-PAP	Precursor/intermediate	$C_{20}H_9F_{34}O_4P$
Perfluorooctane sulfonamido acetate	FOSAA	Precursor/intermediate	$C_{10}H_4F_{17}NO_4S$
N-ethyl perfluorooctane sulfonamido acetate	*N*-EtFOSAA	Precursor/intermediate	$C_{12}H_8F_{17}O_4NS$
N-methyl perfluorooctane sulfonamido acetate	*N*-MeFOSAA	Precursor/intermediate	$C_{11}H_6F_{17}O_4NS$
6:6 Perfluoroalkyl phosphinate	6:6 PFPiA	PFAA	$C_{12}HF_{26}O_2P$
6:8 Perfluoroalkyl phosphinate	6:8 PFPiA	PFAA	$C_{14}HF_{30}O_2P$
8:8 Perfluoroalkyl phosphinate	8:8 PFPiA	PFAA	$C_{16}HF_{34}O_2P$

Table S2. Depth and estimated deposition year in each sediment core layer

Core DWB		Core LD	
Depth (cm)	Year of deposition	Depth (cm)	Year of deposition
0.5	2021	1	2019
1	2020	2	2017
6	2016	4	2014
11	2012	10	2006
16	2007	16	1999
21	2003	22	1991
26	1998	28	1983
31	1994	34	1975
36	1989	40	1967
41	1985	46	1960
46	1981	55	1948
51	1976	61	1940
56	1972		
61	1967		
66	1963		
71	1958		
76	1954		
81	1949		
86	1945		

Table S3. TOC content (‰) in the studied sediment samples

River outlet		NSCS		Core DWB		Core LD	
O1	13.56	P1	8.05	DWB-0.5	10.52	LD-1	9.08
O2	10.84	P3	7.71	DWB-1	9.59	LD-2	8.03
O3	11.68	P5	9.07	DWB-6	8.49	LD-4	8.28
O4	10.78	P7	11.63	DWB-11	9.29	LD-10	8.87
O5	13.07	P10	7.60	DWB-16	8.04	LD-16	8.18
O6	12.66	P13	6.33	DWB-21	7.76	LD-22	9.48
O7	11.59	P16	7.21	DWB-26	7.33	LD-28	10.28
O8	15.25	P20	6.06	DWB-31	7.55	LD-34	7.63
		P22	8.07	DWB-36	6.63	LD-40	9.43
		P26	9.62	DWB-41	6.59	LD-46	9.62
		P28	7.85	DWB-46	5.23	LD-55	8.61
		P33	0.42	DWB-51	6.81	LD-61	7.53
		P37	9.13	DWB-56	6.08		
		P42	10.57	DWB-61	6.89		
		P47	8.33	DWB-66	7.16		
		P52	8.85	DWB-71	6.15		
		P54	8.03	DWB-76	5.27		
		P56	9.06	DWB-81	5.40		
		P59	1.25	DWB-86	6.06		
		P60	7.21				
		P63	6.66				
		P65	9.40				

Table S4. Mass spectrometer parameters for the analysis of PFAS

Compound	Surrogate	Parent ion (*m/z*)	Product ion (*m/z*)	DP (V)	EP (V)	CE (V)	CXP (V)
PFBA	$^{13}C_4$-PFBA	213.2	168.8	-80	-10	-12	-15
PFPeA	$^{13}C_4$-PFBA	262.9	218.8	-60	-10	-12	-19
PFHxA	$^{13}C_5$-PFHxA	312.8	268.8	-75	-10	-12	-19
		312.8	119.0	-75	-10	-26	-13
PFHpA	$^{13}C_4$-PFHpA	363.0	318.9	-55	-10	-14	-23
		363.0	168.9	-55	-10	-24	-13
PFOA	$^{13}C_8$-PFOA	412.9	368.9	-75	-10	-14	-25
		412.9	168.8	-75	-10	-24	-13
PFNA	$^{13}C_9$-PFNA	462.7	419.1	-50	-10	-14	-37
		462.7	168.8	-50	-10	-26	-15
PFDA	$^{13}C_6$-PFDA	513.0	469.0	-90	-10	-16	-41
		513.0	218.8	-90	-10	-26	-15
PFUnDA	$^{13}C_7$-PFUnDA	562.9	518.9	-70	-10	-16	-39
		562.9	269.0	-70	-10	-26	-31
PFDoDA	$^{13}C_2$-PFDoDA	612.8	569.0	-80	-10	-18	-41
		612.8	168.8	-80	-10	-34	-15
PFTeDA	$^{13}C_2$-PFTeDA	713.0	669.1	-90	-10	-20	-47
		713.0	168.9	-90	-10	-36	-15
PFHxDA	$^{13}C_2$-PFTeDA	813.0	769.0	-115	-10	-20	-55
		813.0	168.9	-115	-10	-40	-17
PFOcDA	$^{13}C_2$-PFTeDA	912.8	869.0	-130	-10	-22	-23
		912.8	268.9	-130	-10	-38	-19
PFBS	$^{13}C_3$-PFBS	299.1	79.9	-150	-10	-76	-13
		299.1	98.8	-150	-10	-40	-1
PFPeS	$^{13}C_3$-PFBS	348.9	79.9	-20	-10	-76	-9
		348.9	98.9	-20	-10	-40	-15
PFHxS	$^{13}C_3$-PFHxS	398.9	79.8	-195	-10	-110	-7
		398.9	99.0	-195	-10	-42	-9
PFHpS	$^{13}C_3$-PFHxS	448.9	98.8	-30	-10	-92	-9
		448.9	79.9	-30	-10	-88	-13
PFOS	$^{13}C_8$-PFOS	498.9	79.8	-180	-10	-100	-9
		498.9	98.7	-180	-10	-94	-13
PFNS	$^{13}C_8$-PFOS	548.9	79.9	-45	-10	-118	-11
		548.9	99.0	-45	-10	-130	-47
PFDS	$^{13}C_8$-PFOS	598.8	79.9	-195	-10	-126	-11
		598.8	98.9	-195	-10	-104	-11
HFPO-DA	$^{13}C_8$-PFOA	329.3	168.9	-85	-10	-20	-25
		329.3	118.9	-85	-10	-44	-11
6:2 Cl-PFESA	$^{13}C_8$-PFOS	530.9	350.8	-55	-10	-16	-25
		530.9	98.9	-55	-10	-46	-21
8:2 Cl-PFESA	$^{13}C_8$-PFOS	630.6	450.8	-75	-10	-40	-53
		630.6	98.9	-75	-10	-34	-43
ADONA	$^{13}C_8$-PFOA	376.8	250.8	-30	-10	-20	-23
		376.8	309.0	-30	-10	-20	-23
PFECHS	$^{13}C_8$-PFOS	461.0	381.0	-70	-10	-38	-17
		461.0	256.0	-70	-10	-40	-55
Cl-PFOS	$^{13}C_8$-PFOS	514.8	80.1	-30	-10	-116	-7
		514.8	98.8	-30	-10	-106	-13
6:2 H-PFESA	$^{13}C_8$-PFOS	496.9	316.9	-55	-10	-36	-21
		496.9	296.9	-55	-10	-44	-23

8:2 H-PFESA	$^{13}C_8$-PFOS	597.0	416.9	-55	-10	-40	-21
		597.0	330.9	-55	-10	-50	-29
OBS	$^{13}C_8$-PFOS	603.0	108.0	-85	-10	-75	-10
		603.0	171.9	-85	-10	-90	-11
NTf2	$^{13}C_3$-PFBS	279.9	146.9	279.9	-10	-57	-9
		279.9	77.9	279.9	-10	-34	-7
4:2 FTS	$^{13}C_2$-6:2 FTS	326.8	80.8	-40	-10	-50	-13
		326.8	307.2	-40	-10	-34	-5
6:2 FTS	$^{13}C_2$-6:2 FTS	426.9	406.7	-35	-10	-32	-19
		426.9	80.9	-35	-10	-78	-7
8:2 FTS	$^{13}C_2$-6:2 FTS	526.9	506.9	-25	-10	-38	-7
		526.9	80.9	-40	-10	-35	-9
10:2 FTS	$^{13}C_2$-6:2 FTS	626.9	606.8	-25	-10	-44	-51
		626.9	80.9	-25	-10	-112	-9
FPrPA	$^{13}C_2$-FOUEA	240.9	63.0	-30	-10	-10	-27
		240.9	39.0	-30	-10	-34	-9
FPePA	$^{13}C_2$-FOUEA	340.9	236.7	-50	-10	-20	-13
		340.9	217.0	-50	-10	-32	-13
FHpPA	$^{13}C_2$-FOUEA	441.0	336.9	-30	-10	-16	-37
		441.0	316.8	-30	-10	-28	-19
6:2-diPAP	$^{13}C_2$-PFDoDA	789.0	78.9	-5	-10	-130	-9
		789.0	443.0	-5	-10	-30	-13
6:2/8:2-di PAP	$^{13}C_2$-PFDoDA	888.9	78.8	-10	-10	-124	-35
		888.9	442.8	-10	-10	-30	-19
8:2 di-PAP	$^{13}C_2$-PFDoDA	988.9	78.9	-5	-10	-126	-13
		988.9	542.9	-5	-10	-32	-19
FOSAA	$^{13}C_2$-PFDA	555.9	497.8	-15	-10	-40	-29
		555.9	418.9	-15	-10	-36	-31
N-EtFOSAA	$^{13}C_2$-PFDoDA	584.0	418.9	-20	-10	-26	-11
		584.0	525.7	-20	-10	-30	-35
N-MeFOSAA	$^{13}C_2$-PFDoDA	570.0	418.7	-10	-10	-30	-35
		570.0	482.9	-10	-10	-22	-21
6:6 PFPi	$^{13}C_2$-PFDoDA	701.0	62.8	-60	-10	-128	-9
		701.0	100.8	-60	-10	-130	-11
6:8 PFPi	$^{13}C_2$-PFDoDA	801.0	500.7	-15	-10	-76	-25
		801.0	400.6	-15	-10	-76	-51
8:8 PFPi	$^{13}C_2$-PFDoDA	900.9	500.9	-10	-10	-84	-25
		900.9	62.8	-10	-10	-130	-11

Table S5. Details on the recoveries and method quantification limits (MQLs)

Compound	Recovery (%)	MQL (pg/g dw)
PFBA	82%	20
PFPeA	89%	10
PFHxA	80%	5
PFHpA	96%	2
PFOA	82%	2
PFNA	85%	5
PFDA	64%	2
PFUnDA	80%	2
PFDoDA	72%	2
PFTeDA	68%	2
PFHxDA	67%	2
PFOcDA	87%	5
PFBS	98%	5
PFPeS	69%	5
PFHxS	69%	5
PFHpS	74%	5
PFOS	62%	5
PFNS	60%	5
PFDS	67%	5
Cl-PFOS	58%	5
ADONA	75%	2
6:2 Cl-PFESA	61%	2
8:2 Cl-PFESA	72%	5
HFPO-DA	98%	5
PFECHS	80%	2
6:2 H-PFESA	72%	2
8:2 H-PFESA	64%	5
OBS	74%	5
NTf2	96%	75
4:2 FTS	69%	5
6:2 FTS	68%	10
8:2 FTS	60%	2
10:2 FTS	76%	5
FPrPA	77%	10
FPePA	63%	5
FHpPA	78%	5
6:2-diPAP	72%	5
6:2/8:2-di PAP	82%	2
8:2 di-PAP	79%	5
FOSAA	74%	5
N-EtFOSAA	77%	5
N-MeFOSAA	84%	5
6:6 PFPiA	86%	5
6:8 PFPiA	76%	5
8:8 PFPiA	82%	5

Table S6. Spearman correlation results on PFAS detected in more than half of the surface sediment samples collected from the northern South China Sea

Compound	PFHxA	PFHpA	PFOA	PFNA	PFDA	PFUnDA	PFDoDA	PFHxS	PFOS	6:2 Cl-PFESA	6:2 H-PFESA
PFHxA	1.000	0.829*	-0.227	0.000	-0.150	-0.188	-0.314	-0.429	-0.442	-0.357	-0.714
PFHpA	0.829*	1.000	0.049	-.810*	0.552	0.478	-0.033	-0.333	0.033	0.176	-0.500
PFOA	-0.227	0.049	1.000	0.573	0.191	0.473*	0.511	-0.046	0.587**	0.326	-0.147
PFNA	0.000	-0.810*	0.573	1.000	0.055	0.300	-0.103	-0.286	-0.282	-0.370	-0.300
PFDA	-0.150	0.552	0.191	0.055	1.000	0.765**	0.461	0.467	0.203	-0.026	-0.109
PFUnDA	-0.188	0.478	0.473*	0.300	0.765**	1.000	0.611*	0.308	0.478*	0.230	0.406
PFDoDA	-0.314	-0.033	0.511	-0.103	0.461	0.611*	1.000	0.545	0.664**	0.522	0.214
PFHxS	-0.429	-0.333	-0.046	-0.286	0.467	0.308	0.545	1.000	0.270	-0.035	0.250
PFOS	-0.442	0.033	0.587**	-0.282	0.203	0.478*	0.664**	0.270	1.000	0.789**	0.336
6:2 Cl-PFESA	-0.357	0.176	0.326	-0.370	-0.026	0.230	0.522	-0.035	0.789**	1.000	0.318
6:2 H-PFESA	-0.714	-0.500	-0.147	-0.300	-0.109	0.406	0.214	0.250	0.336	0.318	1.000

* represents $p < 0.05$ (two-tailed test)

** represents the $p < 0.01$ (two-tailed test)

Table S7. Toxicity data of 6:2 Cl-PFESA and 6:2 H-PFESA predicted by ECOSAR model [12]

Chemical	Organism	Duration	End Point	Concentration (mg/L)
6:2 Cl-PFESA	Fish	96h	LC50	5.34
	Daphnid	48h	LC50	4.09
	Green Algae	96h	EC50	10.51
	Fish		ChV	0.74
	Daphnid		ChV	0.92
	Green Algae		ChV	5.36
	Fish (SW)	96h	LC50	6.85
	Mysid	96h	LC50	0.57
	Fish (SW)		ChV	5.39
	Mysid (SW)		ChV	0.02
6:2 H-PFESA	Fish	96h	LC50	33.53
	Daphnid	48h	LC50	23.59
	Green Algae	96h	EC50	42.66
	Fish		ChV	4.22
	Daphnid		ChV	4.18
	Green Algae		ChV	18.01
	Fish (SW)	96h	LC50	42.80
	Mysid	96h	LC50	6.59
	Fish (SW)		ChV	19.19
	Mysid (SW)		ChV	0.29

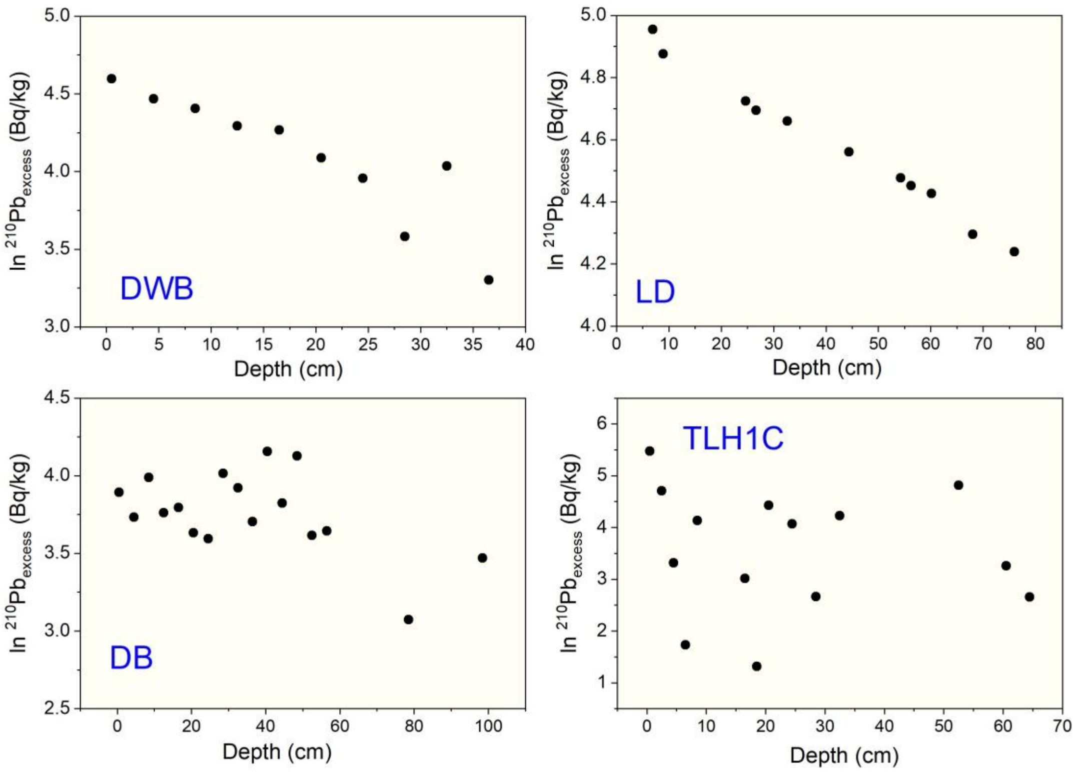

Figure S1. ln $^{210}Pb_{ex}$ in different slices of sediment cores

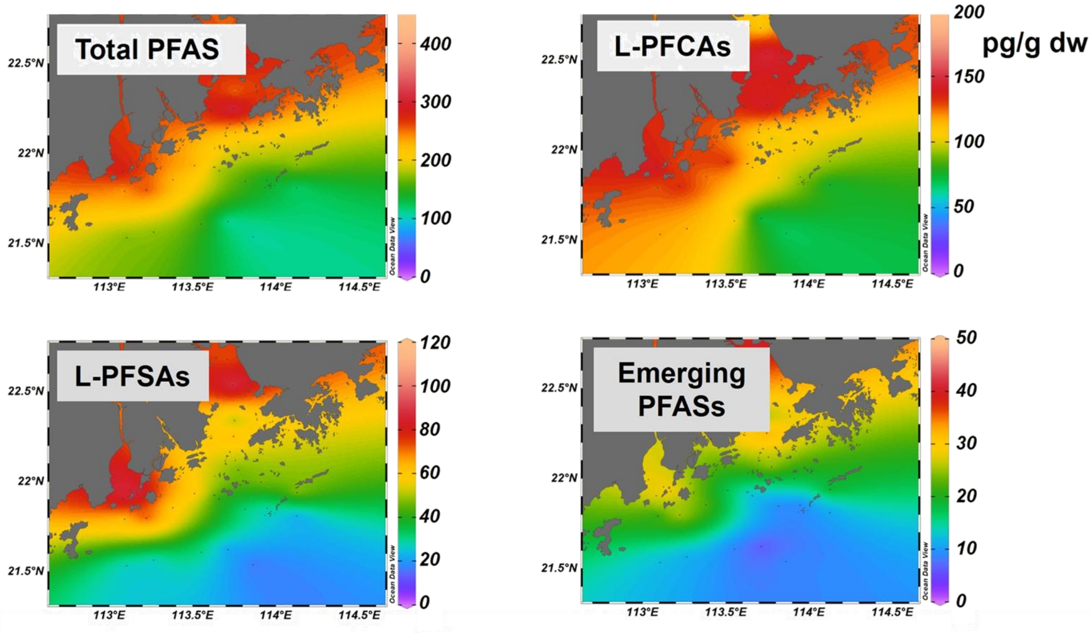

Figure S2. Spatial distribution of PFAS in surface sediment samples collected from the NSCS

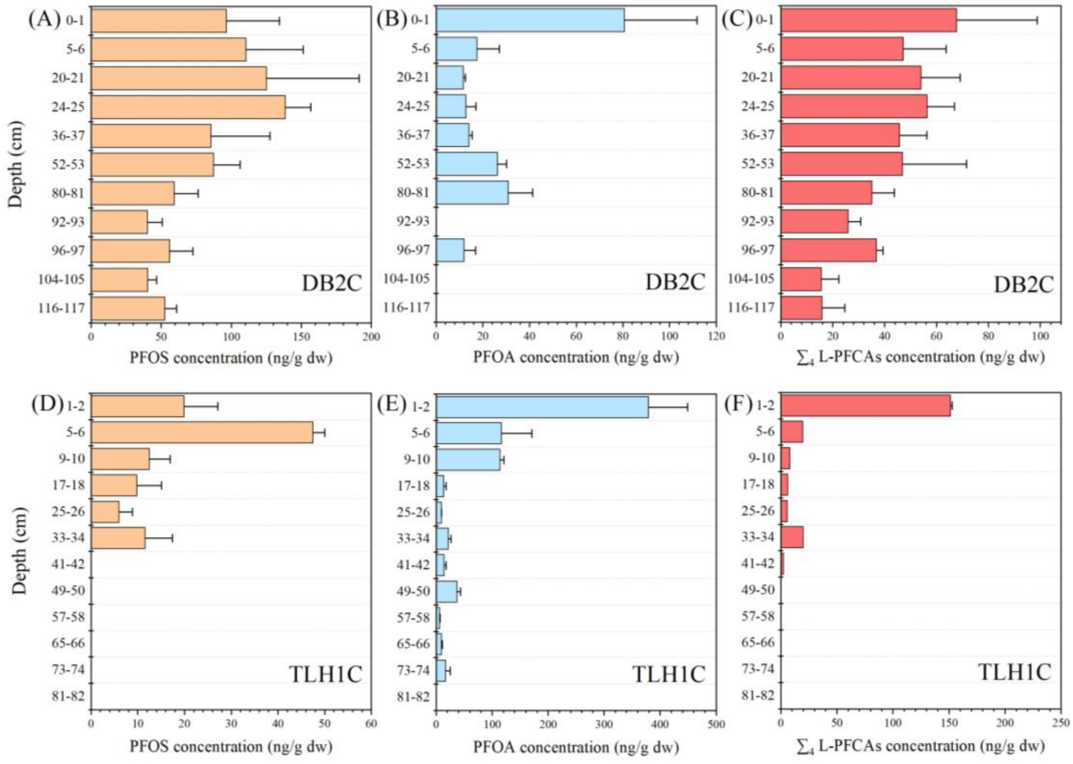

Figure S3. Concentration of PFOS, PFOA, and $\sum_4$ L-PFCAs in sediment cores DB2C (A, B, C) and TLH1C (D, E, F)

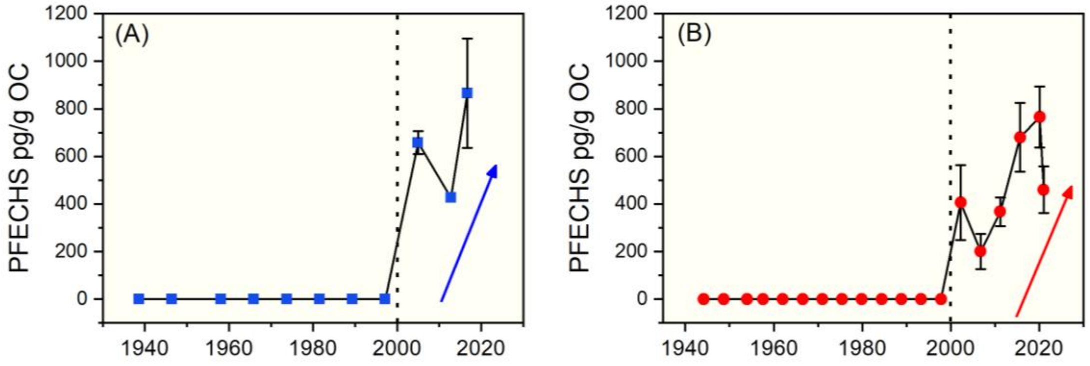

Figure S4. Concentration of PFECHS in sediment cores LD (A) and DWB (B)

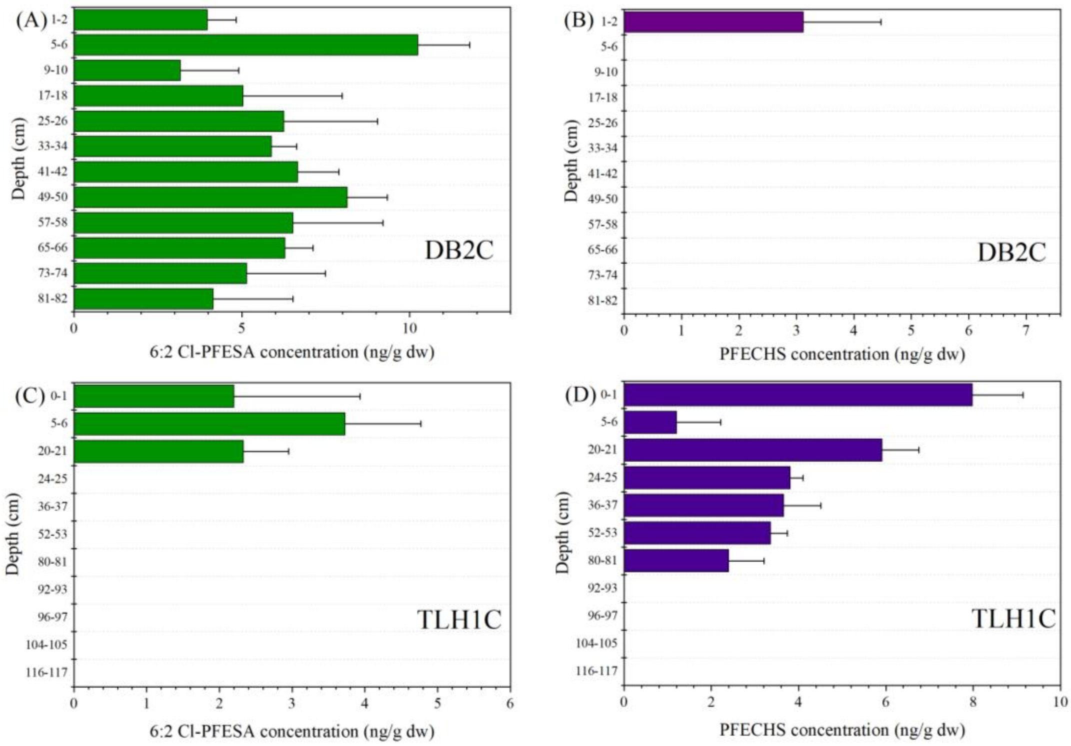

Figure S5. Concentration of 6:2 Cl-PFESA and PFECHS in sediment cores DB2C (A, B) and TLH1C (C, D)

References

1. Nomura, R.; Inoue, M., Comparison of different radionuclide dating models for estimating the ages of sediments from Lakes Shinji-ko and Nakaumi, Japan. *Laguna* **2019,** *26*, 3-26.

2. Blais, J. M.; Kalff, J.; Cornett, R. J.; Evans, R. D., Evaluation of 210 Pb dating in lake sediments using stable Pb, Ambrosia pollen, and 137 Cs. *Journal of Paleolimnology* **1995,** *13*, 169-178.

3. Wang, Q.; Ruan, Y.; Jin, L.; Lu, G.; Ma, L.; Yeung, L. W. Y.; Wang, W.; Lam, P. K. S., Oysters for legacy and emerging per- and polyfluoroalkyl substances (PFASs) monitoring in estuarine and coastal waters: Phase distribution and bioconcentration profile. *Science of the Total Environment* **2022,** *846*, 157453.

4. Wang, Q.; Ruan, Y.; Jin, L.; Tao, L. S. R.; Lai, H.; Li, G.; Yeung, L. W. Y.; Leung, K. M. Y.; Lam, P. K. S., Legacy and emerging per- and polyfluoroalkyl substances in a subtropical marine food web: Suspect screening, isomer profile, and identification of analytical interference. *Environmental Science & Technology* **2023,** *57*, (22), 8355-8364.

5. European Commission, **2013**, Directive 2013/39/EU of the European Parliament and of the Council of August 23 2013. (https://eur-lex.europa.eu/legal-content/EN/TXT/PDF/?uri=CELEX:32013L0039&from=EN, accessed on 14 Feb 2025)

6. Italian Parliament, **2015**, Legislative Decree 172/2015. Gazzetta Ufficialedella Repubblica Italiana. (https://www.gazzettaufficiale.it/eli/id/2015/10/27/15G00186/sg, accessed on 14 Feb 2025)

7. Valsecchi, S.; Conti, D.; Crebelli, R.; Polesello, S.; Rusconi, M.; Mazzoni, M.; Preziosi, E.; Carere, M.; Lucentini, L.; Ferretti, E.; Balzamo, S.; Simeone, M. G.; Aste, F., Deriving environmental quality standards for perfluorooctanoic acid (PFOA) and related short chain perfluorinated alkyl acids. *Journal of Hazardous Materials* **2017,** *323*, 84-98.

8. Wang, Q.; Tsui, M. M. P.; Ruan, Y.; Lin, H.; Zhao, Z.; Ku, J. P. H.; Sun, H.; Lam, P. K. S., Occurrence and distribution of per- and polyfluoroalkyl substances (PFASs) in the seawater and sediment of the South China sea coastal region. *Chemosphere* **2019,** *231*, 468-477.

9. Wang, Q.; Ruan, Y.; Jin, L.; Lin, H.; Yan, M.; Gu, J.; Yuen, C. N. T.; Leung, K. M. Y.; Lam, P. K. S., Tissue-specific uptake, depuration kinetics, and suspected metabolites of three emerging per- and polyfluoroalkyl substances (PFASs) in marine Medaka. *Environmental Science & Technology* **2022,** *56*, (10), 6182-6191.

10. Wang, Q.; Ruan, Y.; Jin, L.; Zhang, X.; Li, J.; He, Y.; Wei, S.; Lam, J. C. W.; Lam, P. K. S., Target, nontarget, and suspect screening and temporal trends of per- and polyfluoroalkyl substances in marine mammals from the South China Sea. *Environmental Science & Technology* **2021,** *55*, (2), 1045-1056.

11. Wang, Y.; Chang, W.; Wang, L.; Zhang, Y.; Zhang, Y.; Wang, M.; Wang, Y.; Li, P., A review of sources, multimedia distribution and health risks of novel fluorinated alternatives. *Ecotoxicology and Environmental Safety* **2019,** *182*, 109402.

12. USEPA, **2024** Ecological Structure Activity Relationships (ECOSAR) Predictive Model. (https://www.epa.gov/tsca-screening-tools/ecological-structure-activity-relationships-ecosar-predictive-model, accessed on 14 Feb 2025)
